# Comparative orotomy of the Archean Superior and Phanerozoic Altaid orogenic systems

**DOI:** 10.1093/nsr/nwac235

**Published:** 2022-10-25

**Authors:** Timothy M Kusky, A M Celâl Şengör

**Affiliations:** Center for Global Tectonics, School of Earth Sciences and State Key Laboratory for Geological Processes and Mineral Resources, China University of Geosciences, Wuhan430074, China; Badong National Observatory and Research Station for Geohazards, Ministry of Education, China University of Geosciences, Wuhan430074, China; Department of Geological Engineering, Middle East Technical University, Ankara 06800, Türkiye; Center for Global Tectonics, School of Earth Sciences and State Key Laboratory for Geological Processes and Mineral Resources, China University of Geosciences, Wuhan430074, China; Eurasia Institute of Earth Sciences, Istanbul Technical University, Istanbul 34469, Türkiye; Department of Geology, Faculty of Mines, Istanbul Technical University, Istanbul 34469, Türkiye

**Keywords:** Altaids, superior province, ophiolite, subduction, strike-slip

## Abstract

We compare and contrast the materials and mechanisms of continental crustal growth in the largest preserved regions of Phanerozoic and Archean juvenile additions to the crust, to test for similarities or differences in the formation of continents through time. We accomplish this through a comparison of map patterns, lithological contents, and structural and metamorphic evolution of the Phanerozoic Altaid orogenic system of Asia, with the Archean Superior Province of the North American Craton, using a method termed comparative orotomy. Both orogenic systems consist of collages of curvilinear belts of eroded arcs, some older continental slivers, and vast tracts of former subduction/accretionary complexes. These contain numerous shreds of portions of the ophiolite suite, slivers of island and continental arcs, and accreted oceanic plateau, all intruded by multiple magmatic suites during or between multiple deformation events, then sliced by large transcurrent fault systems and bent into large oroclinal structures. We make this comparison because the Superior Province is a typical Archean craton that was later, in the Paleoproterozoic, incorporated into the larger North American Craton, and has occupied a central position in several supercontinents (e.g. Kenorland and Nuna, which then formed the core of Columbia, Rodinia, Laurentia and Pangea) during its longevity. Since it is the largest single fragment of Archean continental cratonic lithosphere preserved on Earth, the Superior Province is widely regarded as a testing ground for how Earth's continental crust was formed. Likewise, the Altaids encompass the largest region of crustal growth for the Phanerozoic. Our comparison with the Altaids is needed, as in recent years many myths about how the planet may have responded to higher heat production and flow in the Archean have emerged, because of trends in the science where regional geology is ignored in favor of numerical models, isotopic proxies for assumed models of chemical behavior for crust-forming or tectonic processes, or comparisons with other-worldly bodies that bear little resemblance to our hydrous Earth. Thus, we return to the geological record, and here describe the map patterns, lithological associations, structural patterns and evolution of both the Altaids and Superior Province, showing how comparative tectonics, orotomy, is useful in the absence of meaningful paleomagnetic or biostratigraphic data. We pay particular attention to the style of preservation of disaggregated members of the ophiolite suite (ophirags) and their relationships with other tectonic units, and to the widespread but largely overlooked role of late-stage major transcurrent motions and structural slicing of both Archean and Phanerozoic orogenic systems in defining the present-day architecture of both orogenic systems.

## Teaser

Comparative tectonic analysis of the Archean Superior and Phanerozoic Altaid orogens reveals remarkable similarities pointing to common processes involved in the formation of continental crust over four billion years of Earth history.

## INTRODUCTION

**Figure 1.**
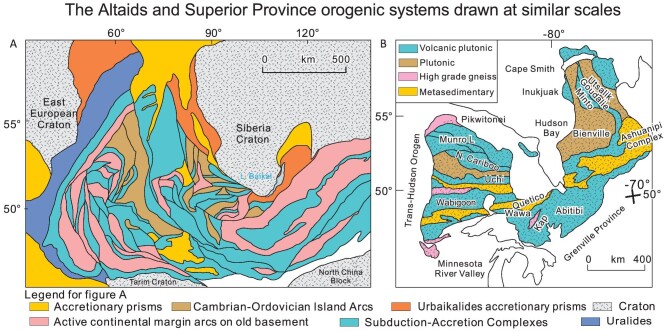
Simplified maps to aid a first-order comparison of the curvilinear tectonic styles in the Paleozoic Altaids (A), and the Archean Superior Province (B), drawn at the same scale (redrawn from Ref. [10]). (A) The Neoproterozoic-Paleozoic Altaid Orogenic System compared with the Archean Superior Province. Note that the types of rocks and tectonic units, and the scales of the curvilinear belts, are remarkably similar between the Archean and Neoproterozoic-Paleozoic orogens. Note also, both orogens have older-consolidated continental-aspect fragments or ribbon continents on their north and south borders, oroclinally bent ribbon continents in their interior, and have an over change in strike of 90° across their width. The Altaids are bordered on the west by the Uralides, then the East European (or Russian) Craton, whereas the Superior Province is bordered on the west by the Trans-Hudson orogen. Detailed maps of the Altaids and Superior orogenic systems are shown in Figs 3 and 6 respectively.

In recent years, a debate has developed in the geoscience community about whether or not ‘modern-style’ plate tectonics operated in the Archean. This debate is based in part on claims that outcrop patterns of different cratons are unlike those produced in the Phanerozoic, and the fuliginous Archean geological record lacks ophiolites and remnants of oceanic crust and lithosphere, which are considered one of the diagnostic indicators of plate tectonics in Phanerozoic orogens, marking places where former oceans have closed. This is rooted in the fact that many geologists still apply the 1972 Penrose definition of ophiolites and insist that to be recognized as a slice of former oceanic lithosphere, the ophiolite must include the full suite grading down from pillow lavas to mixed pillow lavas and dikes, to a sheeted dike complex, into gabbros and layered gabbros, peridotites and dunites, and tectonized mantle harzburgites. Following Şengör and Natal’in's seminal paper in 2004, we here restrict the use of the term ophiolite to this full suite (which is rare even in Phanerozoic orogens), and we use the term ‘ophirag’ for any of the various components of the ophiolite suite that were shredded off from the oceanic crust during their history of formation, attempted subduction, obduction and incorporation into orogens and ultimately the continental crust. In this contribution, we describe and compare the geology and tectonic history of the dominantly Phanerozoic Altaid orogenic system [1] of Asia with the Archean Superior Province [2] of the North American Craton [3–6] (Fig. 1). Comparative orotomy is a branch of tectonics that studies the structure of mountain ranges in comparison with one another against a background of the fundamental processes of orogeny, i.e. any set of phenomena taking place at convergent plate boundaries. These phenomena create structures, called orogens, that collectively build natural tectonic edifices that deviate less from one another than from other tectonic ensembles. Comparative orotomy is, like its zoological counterpart anatomy, a science of dissection, dismemberment and reconstruction. The term orotomy is derived from the Ancient Greek words ὄρος (mountain) and τ$\acute{\varepsilon}$μνω (I cut, dissect) and thus literally means ‘mountain dissection’. Orotomy is a new term introduced in conceptu by Şengör and others [1], and applied here, in nomine, for the first time.

Both the Altaids and the Superior orogenic systems (Fig. 1) contain hundreds to thousands of kilometer-scale belts of magmatic arcs, subduction accretion complexes and older ribbon continents, sliced by strike-slip faults associated with late transtensional and transpressional basins. Within and between these belts, large irregular areas characterized by intense orogenic deformation, typically containing many small ophirags, are intruded by multiple generations of plutonic rocks, and deformed into complex patterns including the classic ‘dome-and-basin’ pattern that typifies deeply eroded arc roots of all ages [7,8]. In this contribution, we show that the overall map patterns, lithological make-up, structural patterns and the characteristic shredding of the former ophiolite suite into the presently preserved ophirags in these orogenic collages, is no different in the Altaids than in the Superior Province, or in other Archean cratons or orogens. In the Altaids, ophiolitic crust is most commonly found as: (i) basement to oceanic arcs; (ii) remnants of former forearcs (forearc ophiolites) and in accretionary wedges (with Mid Ocean Ridge Basalt [MORB] characteristics), both typically deformed in later transcurrent structures; and (iii) strongly attenuated and metamorphosed ophirags in collisional sutures, derived from the above types. We follow this by showing that members of the ophiolite suite (now ophirags) occur in precisely the same tectonic settings in the Superior Province, albeit with a greater proportion of thickened oceanic-plateau-type fragments, leading us to conclude that both the Phanerozoic Altaids and the Archean Superior Province were produced by remarkably similar tectonic processes, and that we can look to the Altaids as a modern analog to the processes that formed the Archean continental crust. This comparison is particularly helpful with the lack of biostratigraphic and paleomagnetic control, which has been so useful in deciphering the complexities of the tectonic evolution of the Altaids, but which is unavailable in the Superior Province and other Archean cratons [6,9,10].

### Definitions and significance of ophiolites and ophirags

The term ‘ophiolite’ was first introduced into the geological literature by Alexandre Brongniart [11], for rock units basically consisting of serpentinite encompassing various minerals, but expanded by him in 1821 [12] and 1827 [13], in descriptions of the Ligurian ophiolites in the Appennines, to include serpentinites with their mineralogically varied included blocks that were associated with gabbros, mafic volcanics and cherts. In Brongniart's definitions, he referred to ophiolite as only the ultramafic part of what was later defined in the Penrose Field Conference participants’ report [14] as an ophiolite, *sensu stricto* (in the terminology of Dewey [15]).

**Figure 2.**
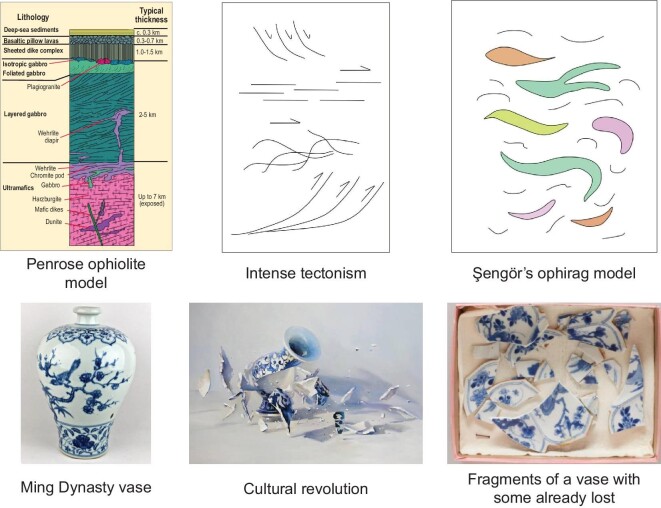
The classic Penrose ophiolite model [14], compared with the ophirag model [28]. As in the ceramic vase, ophirags may start out whole, but become shredded when incorporated into accretionary complexes or suture zones.

One of the most polarizing events in the history of ophiolite studies was the 1972 Geological Society of America (GSA) Penrose Field Conference on Ophiolites [14], where participants assessed the stratigraphy of two of the most unusual (at that time, thought of as ‘complete’ or ‘typical’) large ophiolitic sheets, the Troodos of Cyprus and the Semail of Oman. The participants suggested that ‘typical’ ophiolites (and hence, normal oceanic crust, to which it was being compared) were 5–15 km thick, and consisted of a regular sequence (Fig. 2) grading up from a fault at the base that was related to the emplacement of the ophiolite.

The basal unit of ‘Penrose-type’ ophiolites thus was defined as consisting of harzburgite (olivine + orthopyroxene, +/- chromite), typically with a strong, high-temperature transposed tectonic fabric, forming a mantle harzburgite tectonite (e.g. [16]), and identified as depleted or residual mantle, from which the overlying crustal sequence was derived. In a few ophiolites the basal unit, or a unit underlying the harzburgite, was recognized as lherzolite, interpreted as undepleted mantle. The high-temperature fabrics in the harzburgite tectonite were shown to have formed during the high-temperature flow of the mantle sequence, after it rose and partially melted, then flowed away from the axis of the ridge, constituting the mantle component of sea-floor spreading. Some of the thickest mantle harzburgite tectonite sequences (∼10 km) are preserved in the Semail ophiolite of Oman [17] and the Bay of Islands of Newfoundland [18,19]. The petrologic Moho separates the depleted rocks of the mantle harzburgites below, from the rocks of the crust, derived from the mantle, above. The lowest crustal unit is typically ultramafic cumulates, including pyroxenite, dunite and other olivine + orthopyroxene + clinopyroxene cumulates including wehrlite and websterite, often associated with pods of chromite + olivine. Anorthosite is present in some instances. The seismic Moho lies above these dense cumulates, above which are seismically slower units of interlayered pyroxenite and plagioclase-rich cumulates, passing into a unit of layered gabbro with a typical thickness of ∼1 km. In some Penrose-type ophiolites, such as Semail, the seismic Moho is intruded by multiple gabbroic sills, so the Moho appears repeated in the field.

A unit referred to as isotropic gabbro, up to 5 km thick, overlies the layered gabbro in ‘typical Penrose-type’ ophiolites (Fig. 2). The isotropic gabbro is typically massive but may preserve a weak igneous layering, which in some cases defines a curving trajectory that is thought to have formed by crystallization along the walls of the magma chamber, so can be used to define the magma chamber's shape through detailed mapping (e.g. [20–22]). The upper part of the isotropic gabbro is associated with trondhjemitic dikes, pods and other intrusions, plus may have xenoliths of basalt or diabase, and may be cut by diabasic dikes.

The next unit in a complete ‘Penrose-type’ ophiolite is the famous sheeted dike complex, typically 0.5–2 km thick (Fig. 2) and consisting of complex diabasic, gabbroic, trondhjemitic and silicic dikes, that grade into and show mutually cross-cutting relationships with the underlying gabbro. In the ideal sheeted dike complex (of which there are few), each succeeding diabasic dike intrudes into the center of the previous dike, splitting it in half, and leaving a sequence of dikes that have chill margins only preserved on one side. In these ideal cases, the side of the dike without the chill margin faces towards the paleo-spreading axis. More recent work on ophiolites globally has shown that while these classic sheeted dike complexes do occur, they are present in only 10%–15% of ophiolites globally, and in those, documentation of one-way chill margins is exceedingly rare [23–25] because of the rarity of their formation and difficulty of preservation through deformation and metamorphic recrystallization of fine-grained primary igneous textures. In some ophiolites, the sheeted dike complex is replaced by a sill complex.

The sheeted dikes, which were formed as cracks that filled with magma from the underlying magma chamber, fed the volcanic sequence marking the magmatic top of the ophiolite. The upper magmatic unit is typically marked by pillowed or massive basaltic flows because of their undersea eruption, and many are associated with hyaloclastites or volcanic breccias formed by the implosion of the hot pillows upon their contact with seawater. In some ophiolites, pillow interstices are filled by chert, jasper or metalliferous sulfides, and may be associated with large massive sulfide deposits and geological and biological records of activities around black smoker chimney-type submarine exhalative systems.

Many Penrose- (and other) type ophiolites are overlain by deep-sea sediments. In Phanerozoic oceans these include partly dissolved pelagic limestones, cherts, red clay, carbonates, pyroclastic deposits, sulfides, Mn-rich deposits and biogenic oozes. As the oceanic crust and lithosphere moves across the ocean basin towards a trench, it is typically covered by progressively more continent- or arc-proximal clastic deposits [26], forming a classic ocean-plate-stratigraphic sequence that reflects the specific history of the ocean crust's transport (e.g. [27]).

Following the Penrose definition of ophiolite (see [25] for a review), many geologists became increasingly insistent that to be classified as an ophiolite (and a presumed remnant of some form of oceanic crust) the whole Penrose-type sequence needed to be preserved intact, including the sheeted dike complex. This led to a great backward step in understanding ophiolites, and many studies of what are now recognized to be fragments of oceanic crust and lithosphere, or forearc crust, were dismissed by virtue of their fragmentary nature, or preservation of an ‘incomplete’ sequence [28].

Later, it was shown [24] that only ∼15% of ophiolites preserve sheeted dike complexes, and that their initial formation was critically dependent on the relative balance between extension rate and magmatic supply. If the magmatic supply is too low, no dike complex forms, and the crustal sequence may be very thin, replaced instead by normal faulting that brings up deeper mantle rocks in oceanic core complexes [29,30], in the cases of slow and ultra-slow spreading (relative to magmatic supply). In the opposite extreme, in cases where the magma supply exceeds the spreading rate, a very thick crustal section may result, forming an Icelandic-like plateau that may even emerge above sea level.

### Suprasubduction and other settings

These variations were followed by a new recognition of the diverse tectonic settings in which ophiolitic crust may form (e.g. [25,31]), showing that the early findings by [32–35] and [36], i.e. that many ophiolites form in suprasubduction zone settings, either in arcs, backarcs or forearcs, were correct. It is now widely believed that many ophiolites formed during subduction initiation (at a transform or ridge, see [37]) where older oceanic crust is extended by slab rollback, so that the igneous progression moves from older MORB, to boninite, to Island Arc Tholeiite (IAT) over a few to tens of Ma, a process that is now thought to have formed some of the giant ophiolitic sheets such as Troodos, Semail and Bay of Islands (e.g. [38,39]). However, some recent classification schemes (e.g. [40–42]) may be too encompassing, referring to nearly all kinds of magmatic rocks that form in oceans as ophiolitic. Thus, while the classification of Dilek and Furnes [40] is useful for separating ophiolites that are associated with subduction systems from those that are not, we exclude from our classification of ophiolites those magmatic sequences that clearly formed in island arcs (but not those that island arcs were built upon: the ‘pre-arc spreading’ ophiolites: [36]), oceanic islands (seamounts) and oceanic plateaus, all of which have different igneous stratigraphy, rock types and geochemical signatures from ‘normal’ oceanic crust and lithosphere generated at oceanic spreading centers. These may be associated with subduction in forearcs or backarcs, or may form in the ‘center’ of ocean basins formed by Wilson Cycles and the splitting of older continents, or form in Pacific-type-ocean basins during kinematic reorganization of the plate mosaic (e.g. [43]).

Modern understanding of ophiolites thus incorporates the above knowledge that only the upper and dismembered portions of oceanic lithosphere may be preserved in accretionary orogens (e.g. [44]), and also reflects a new understanding of the relative balance between the relative rates of extension and rates of magmatism available to fill the space created by the ongoing extension [24]. It is also recognized that many if not most ophiolites probably did not form at mid-ocean spreading centers as originally thought and defined in the 1972 Penrose definition, but may reflect a diversity of oceanic extensional environments including mid-ocean ridges, stretched continental margins, extensional forearcs and backarcs, and Iceland-like thickened plateaus (e.g. [25,40]). With these new definitions, ophiolites are now generally regarded as ‘a fragment of oceanic lithosphere emplaced on a continental margin or in an island arc’ [24].

In cases where the rate of extension exceeds the ability of available magmatism to fill the gap created by the extension, oceanic spreading will produce a lithospheric structure where the crust is very thin or absent, and isolated volcanoes of pillow basalts fed by small gabbro intrusions rest directly over depleted mantle peridotite, or form oceanic extensional core complexes ([29]; see Whitney and others [30] for a review). If, in the ocean, these conditions can produce oceanic core complexes where the harzburgitic mantle is directly exposed on the sea floor, then eventually they will be overlain by the ocean-plate stratigraphic (OPS) sequence [27,45]. In stretched continental margins, the lherzolitic sub-continental lithospheric mantle may be juxtaposed with thinned continental crust (‘transitional ophiolites’ of Kusky and others [25]), with mafic intrusions in proportion to the amount of melt available, such as in the modern case of Tahir Asir on the Red Sea coast [25] or in the Platta Nappe in the Alps [45]. In cases where the spreading/extension rate is exactly equal to the rate of magma supply, a ‘classical’ Penrose-type ophiolitic structure will emerge, though it has been found that this is only preserved in ∼15% of extant ophiolites [23,24]. In the final extreme, in cases where the amount of magmatism greatly exceeds the volume created by extension, a very thick crustal section will be produced, generating an Icelandic-like plateau, or a larger Large Igneous Province (LIP) such as the Caribbean oceanic plateau [44,46–48] plus its obducted sliver in the Nicoya Complex in Costa Rica [49]. Superimposed on the above newly recognized basic structure of sea floor that can produce ophiolites is the recognition that these environments that produce oceanic lithosphere (melt-depleted, melt enriched or balanced) may be found at mid-ocean ridges, stretched continental margins or in fore- and backarc settings, with concomitant changes in the chemistry of the magmas, especially related to whether or not they are influenced by slab-derived fluids (e.g. [40]).

### Ophirags

Although dismembered and metamorphosed varieties of ophiolites were recognized by the Penrose [14] committee, it was not until JF Dewey [15] that it became widely recognized that most ophiolites were highly dismembered during the emplacement process, leading to only scattered remnants of different parts of the sequence in mountain belts. Dewey applied the terms ‘ophiolite, *sensu stricto*, and ophiolite, *sensu lato*’ to the complete and the shredded varieties, respectively, interpreting the full sequences as representing very special circumstances of formation and special tectonic configurations leading to their preservation. On the other hand, ophiolites ‘*sensu lato*’ included the widely recognized ophiolitic mélanges and associations such as the ‘slate-diabase’ units of the Tethysides [50]. While this terminology is still used in some cases to this day and applies to many mappable units in orogens of all ages, it still does not adequately characterize, in a single word or definition, the many fragments and scraps of oceanic material found in circum-Pacific-type ‘accretionary’ orogens with coastwise transported crustal slivers and large subduction-accretion complexes, or those of the Altaids or many Archean orogens and cratons. While commonly contentious, it has been recognized since [51] that oceanic-type fragments of former ophiolites are found in many Archean terranes, such as the Slave [52], Superior [44], Kaapvaal [53,54] and many other cratons [23,55,56]. In many cases, only the upper crustal sections of ophiolites may be preserved, a characteristic of Archean ophiolites, which likely were scraped off from a thicker oceanic crustal section [8,44,52,57].

These ideas were brought together in a seminal paper, where Şengör and Natal’in [28] addressed the issue of how to interpret scattered remnants or fragments of former ophiolitic bodies that have been torn apart and shredded during emplacement into accretionary wedges, or onto continents. These authors suggested retaining the term ophiolite for the large coherent sheets that have the ‘complete’ Penrose sequence, but suggested adopting the term ophirag (Fig. 2) for highly dismembered fragments of deep-sea sedimentary deposits, basalts, diabase, gabbros and mafic/ultramafic cumulates, and both lherzolitic and harzburgitic mantle rocks, that may have originated as pieces of oceanic crust and mantle, seamounts, forearcs, backarcs, arcs or stretched continental margins (see the classification of ophirags according to Kusky and others [23,25]), all later incorporated into continents. The term ‘ophirag’, according to Şengör and Natal’in [28], is derived from the Greek ϕ$\acute{\iota}$δι (for serpent or snake) and σχ$\acute{\iota}$ζα (for tattered, shred or sliver). We adopt the terminology of Dewey [15] and Şengör and Natal’in [28] for the remainder of this work. It is important to note that ophirags, while they do not contain the whole ophiolite suite, are like broken pieces of valuable vase (Fig. 2); they still contain enough information to be able to interpret their original formation environment as part of the oceanic lithosphere.

### Ophiolites as markers of places where oceans have closed

Much of the early work on ophiolites done in the Alpine-Himalayan sectors of the Tethysides led to the idea that ophiolites represent good markers of the places where oceans have closed, since many of these examples are distributed in linear belts, with associated ophiolitic mélanges, separating tectonic units of different character (e.g. [15]). The observations behind this idea can be traced back to at least Suess in 1904 [58], who noticed chaotic blocks in sheared scaly matrixes beneath large thrust nappes in the Alps, and suggested, by analogy with glacial moraines, that these were blocks brought up from the deep along curved thrust faults. He further noted the association of ultramafic rocks with these thrust faults, noting that the thrusts must be bringing rocks to the surface from much deeper in the Earth. This concept was later followed by Gustav Steinmann, who described ‘jumbled rock masses (Aufbruchszone)’ at the bottom of the Austroalpine nappe system in eastern Switzerland [59,60], which were in many places ‘reduced to mere slivers’ [61]. Steinmann is best remembered for his association of radiolarites and deep-sea muds, with gabbros and serpentinized peridotites (comprising the Steinmann Trinity; [62–64]), and he suggested [59] that these rocks were formed at depth and uplifted by the medial Cretaceous during the Alpine Orogeny. These early observations, to which the association with pillow lavas was later recognized, as summarized well by [65] and [66], laid the foundations for what we recognize today as the Alpine sutures, demarcated by ophiolitic mélanges and ophiolites.

While the above concepts evolved under the influence of the geosynclinal theory, Argand [67], who was a follower of Alfred Wegener's continental drift idea, recognized that the ‘geosyncline’ of the Alps was once an extensional basin, and that the rocks now regarded as ophiolites would have formed in the great depths of this ocean and been mixed with debris flows that slumped into the basin from the sides. Later, Stille, in 1939 [68] suggested that rocks now recognized as ophiolites formed during the initial subsidence phases of ‘geosynclines’ and provided detailed descriptions for the formation of ophiolites at the time. Many of these early studies led to the great syntheses of Hess in 1955 [51], who recognized that ophiolites consist of a mafic/ultramafic association that is commonly associated with deep-sea sediments, that they are found only in orogenic belts, and that while many occurred as large tabular bodies, most formed scattered, smaller, highly deformed fragments in orogens. These ideas were readily adapted later into the plate tectonic paradigm, although 70 years after Hess's seminal paper, one of his most significant observations is still not widely appreciated. While Hess noted that Alpine-type peridotites were known predominantly from Alpine-type mountain belts, he pointed out that they also ‘appear to occur ubiquitously throughout the earliest Precambrian rocks’ ([51], p. 394).

### Structure and evolution of the Altaids

The Altaids represent the world's best Phanerozoic analog for Archean greenstone/gneiss-tonalite complexes (Fig. 1; see [69–71]). They are characterized by an overall equant map aspect brought about by extensive strike-slip stacking during their evolution; vast terrains of flysch complexes dominated by turbidites of both distal and proximal facies, in places intermingled with deep-sea carbonates and radiolarian cherts, stacked together with basalts; in some other localities larger basaltic massifs, interpreted as fragments of former oceanic plateau, are capped by shallow water carbonates; the vast flysch complexes contain shreds of various members of the ophiolite pseudostratigraphy, termed ophirags, plus knockers of carbonate rocks and, rarely, sandstones and felsic rocks. In many areas, these highly deformed rocks are covered by shallow water carbonates, clastics and/or entirely terrestrial clastic cover plus rare evaporites, and intruded by a wide spectrum of igneous rocks ranging from arc gabbros through tonalites, monzonites, granodiorites to granites, the last appearing mainly towards the end of the orogenic deformation. In places, there are sparse syenitic intrusives associated with trachytes and basaltic lava flows, including the immense Siberian traps of the latest Permian (covering nearly 7 million square kilometers). All of these rocks have been suggested to have been produced in large, Makran-sized subduction-accretion wedges invaded progressively by arc magmatism and thus, in time, themselves turning into arc massifs. Behind them in most places are older, Precambrian continental slivers, which had functioned as the original arc massifs in front of which the subduction-accretion complexes had begun to accumulate. Especially in the Central Altaids, the Kazakhstan and Altay parts, there are ensimatic massifs that functioned as backstops. Dominantly right-lateral strike-slip faulting accompanied subduction-related activity throughout the Altaid evolution, disrupting the orderly migration of magmatism from arc central lines towards the external parts by removing well-developed subduction-accretion prisms from the fronts of some of the arcs and thus exposing them to renewed subduction magmatism, giving the impression of a migration ‘backwards’ towards the original arc axis. Whether any subduction-erosion has happened anywhere in the Altaids is hard to tell, but given the large amounts of accreted materials at all times during the Altaid evolution it is unlikely to have been significant. In some segments there are relicts of what are interpreted to be former backarc basins that created ‘double sutures’; in others, strike-slip faulting created pull-aparts ranging in size from small alkalic magmatic nodes to vast and deep sedimentary basins such as those of Junggar and Nadym (in Nadym, sedimentary thicknesses may have reached 18 km!) The larger of these formed exclusively during the final phases of the assembly of the Altaid collage.

One peculiarity of the Altaid orogenic collage is the paucity of any sedimentary facies belts that would allow one to follow the main corpus of the orogen along the strike and identify its trendlines. Instead, the sharp magmatic fronts of arcs provide excellent structural markers to follow the trend of the original subduction-related orogenic belt that was later disrupted by extensive strike-slip faulting and ongoing orogeny [72–76]. This method allows one to identify what Şengör *et al*. [6] termed the essential units in an orogenic collage, i.e. those entities that formed during the subduction activity and characterize the various kinds of arcs. When the essential units are disrupted during or after the orogeny by diverse kinds of faults, but mainly by large offset strike-slip faults, indeed entire keirogens (defined as a broad strike-slip shear zone with more than one strike-slip fault [77]), accidental units come into being. Essential units may be characterized by a limited set of rock types disposed in regularly arranged structures such as backarc basins (of both extensional and shortening types), arc massifs (that may be extensional, neutral or shortening) and forearcs (which may be formed entirely from trapped ophiolites with small accretionary complexes or from vast subduction-accretion prisms with one or more forearc sedimentary basins). Accidental units, on the other hand, appear as haphazard slices or slivers of diverse rock types bounded by a multitude of fault belts of multifarious types. That is why ‘terrane’ methodology (and terminology) fails entirely, as it throws into one basket both essential and accidental units that give different messages about the evolution of an orogenic system; its terminology renders a mountain belt a patchwork quilt, each patch carrying most commonly a geologically wholly uninformative name. It should therefore be avoided.

### Ophirags of the Altaids

In the Altaids, ophiolitic crust is most commonly found as: (i) basement to oceanic arcs; (ii) remnants of former forearcs (forearc ophiolites) and in accretionary wedges (with MORB characteristics), both typically deformed in later transcurrent structures; and (iii) strongly attenuated and metamorphosed ophirags in collisional sutures, derived from the above types.

**Figure 3.**
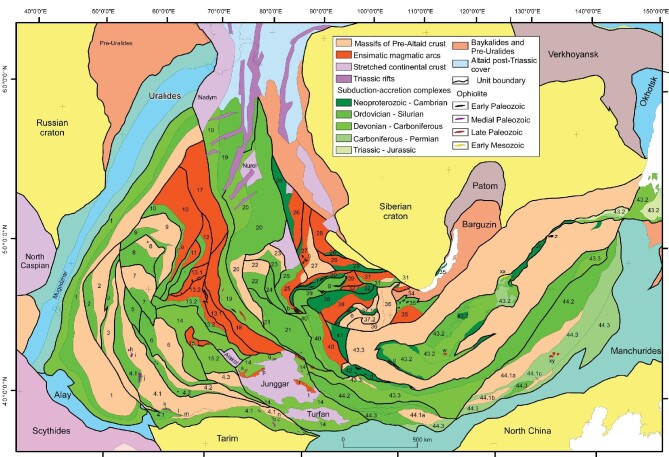
Tectonic map of the Altaids (from Şengör *et al*. [1,70]). First-order tectonic units of the Altaids, including ophiolite and ophirag occurrences within them. The map has an equidistant conical projection, with the central meridian at 95°, and the standard parallels are 1:15.0° and 2:85.0°. The latitude of origin is 30.0°. Arabic numerals, and the lowercase letters attached to some of them, identify the first-order tectonic units of the Altaids (for the definition of first- and second-order tectonic units, see Şengör and Natal’in [73] and for the new nomenclature (essential and accidental units), see Şengör [38,39,72–74,76] and Lom *et al*. [75]). Lowercase letters identify individual ophiolite and ophirag occurrences. First-order tectonic units: 1, Valerianov-Chatkal; 2, Turgay; 3, Baykonur-Talas; 4.1, Djezkazgan-Kirgiz; 4.2, Jalair-Naiman; 4.3 or 16, Borotala; 5, Sarysu; 6, Atasu-Mointy; 7, Tengiz; 8, Kalmyk Kol–Kökchetav; 9, Ishim-Stepnyak; 10, Ishkeolmes; 11, Selety; 12, Akdym; 13.1, Boshchekul-Tarbagatay; 13.2, Bayanaul-Akbastau; 14, Tekturmas; 15, Junggar-Balkhash; 16 or 4.3, Borotala; 17, Tar-Muromtsev; 18, Zharma-Saur; 19, Ob-Zaisan-Surgut; 20, Kolyvan-Rudniy Altay; 21, Gorny Altay; 22, Charysh-Chuya-Barnaul; 23, Salair-Kuzbas; 24, Anuy-Chuya; 25, Eastern Altay; 26, Kozhykhov; 27, Kuznetskii Alatau; 28, Belyk; 29, Kizir-Kazyr; 30, North Sayan; 31, Utkhum-Oka; 32, Ulugoi; 33, Gargan; 34, Kitoy; 35, Dzhida; 36, Darkhat; 37, Sangilen; 38, Eastern Tannuola; 39, West Sayan; 40, Kobdin; 41, Ozernaya; 42, Han-Taishir; 43, Tuva-Mongol; 43.1, Tuva-Mongol Arc Massif; 43.2, Khangay-Khantey; 43.3, South Mongolian; 44, South Gobi. Selected ophiolite and ophirag occurrences in the Altaids (many smaller occurrences could not be shown because of the scale of the map): a, Kuyanbai; b, Eastern Altay ophirags; c, Kuznetskii Alatau basement; d, Han-Taishir (transliterated as Khantaishir in some publications); e, Agardag; f, Boruss mélange with ophirags; g, Kurtushiba; h, Kara-Archa ophirags; i, Kenkol; j, Toluk; k, Karachi-Karakty; l, Karadzhorgo; m, Archaly; n, Youshugou ophirags; o, South Tien Shan ophirags; p, Dabut; q, Honguleleng; r, Karameili; s, Mayila; t, Aermentai; u, Bayankhongor; v, Dzida; w, ophirags in Khangai-Khantei; x, Kulinda; y, Molodovsk; z, Gorbits; xx, Ust-Trua; xy, Hegeshan. These ophiolite and ophirag occurrences are reviewed in detail by Şengör and Natal’in [75].

**Figure 4.**
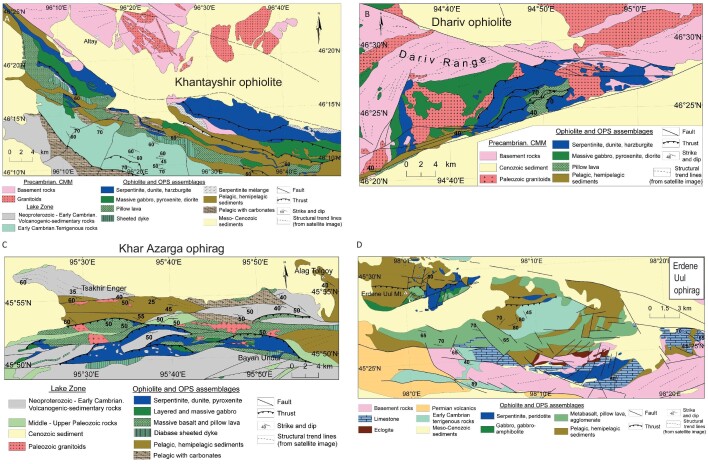
Geological maps of the (A) Khantayshir, (B) Dhariv, (C) Khar Asarga and (D) Erdene Uul ophiolite fragments and ophirags. Maps are all adapted from Khukuudei *et al*. [78].

Khukuudei *et al*. [78] have recently synthesized geological maps and data concerning the ophiolites, ophirags and OPS sequences of western Mongolia, and we highlight the features of a few of the better-documented ones here, based on that study. In the southern Lake Zone of the Khukuudei *et al*. classification (corresponding to tectonic units 40, Kobdin; 41, Ozernaya; 42, Han-Taishir; zones of Fig. 3), the main ophiolites are in nappes thrust over the Precambrian crystalline basement of the Central Mongolian microcontinent, and include the large Agardag-Tes, Khantayshir and Dhariv massifs, all ∼570 Ma in age. There are numerous smaller dismembered ophirags present in most of the belts designated as subduction/accretionary complexes in Fig. 3. The ophiolites generally include dismembered sections of serpentinite, dunite, harzburgite, gabbro, anorthosite, pyroxenite, diorite, sheeted dikes, pillow lavas and serpentinite mélange, overlain by chert, shale, siltstone, tuffs and conglomerate [78]. Below, we describe major features of the Khantayshir, Dhariv, Khar Azarga and Erdene Uul ophiolites/ophirags (Fig. 4), as each highlights different degrees of preservation of, and dismemberment from, original relationships, and are useful for comparison with similar-scale examples from the Superior Province, as described below.

The Khantayshir ophiolite covers ∼260 km^2^ and is one of the most complete ophirags of the Lake Zone (Fig. 4A), including serpentinized harzburgite and minor dunite dikes and pods (including podiform chromitites) at its base, to overlying Ediacarian-Lower Cambrian siltstones and chert. A 100–150 m thick transition zone has pyroxenites at its base and gabbros above, although they are mutually gradational, and overlain by layered cumulate dunites, wehrlites, websterites and pyroxenites, and then interlayered pyroxenites and layered gabbros [78]. The next unit is a 250–300 m thick massive-to-brecciated gabbro, capped by sheeted dikes with gabbro screens. This unit passes up into ∼200 m of pillowed lavas and micro-gabbros. Chemically, the ophiolite includes MORB tholeiites, boninites and a weakly developed calc-alkaline andesitic basalt-andesite series [79], suggesting that the ophiolite formed in a forearc setting whereby the forearc represents trapped mid-ocean ridge basement. It is overlain by pelagic cherty shales, red-bedded cherts, volcanogenic epiclastic rocks and archaeocyathid limestones [78].

The Dhariv ophiolite (Fig. 4B) occurs in a series of steeply south-dipping thrust sheets with individual thrust sheets dominated by serpentinized dunite and harzburgite, massive gabbro and pyroxenite with minor diorite, pillow lavas, or pelagic-hemipelagic sedimentary rocks (Fig. 4B). Geochemical studies of the gabbro and pyroxenite [80] suggest that the Dhariv ophiolite has Normal-Mid Ocean Ridge Basalt (N-MORB) characteristics, although it is overlain by a tuffaceous, chert, pyroclastic and tephra-bearing sequence, suggesting it formed in close proximity to an island arc. Supporting formation in a suprasubduction zone setting, the sheeted dikes of the Dhariv ophiolite have a calc-alkaline affinity, and range in composition from basalt, to andesite, dacite, rhyolite and trondhjemite [80]. Here, too, we have a MORB crust trapped in a forearc setting.

The Khar Azarga ophirag (Fig. 4C) is generally similar to the Khantayshir and Dhariv ophiolites, but is highly dismembered, up to 4 km wide and 50 km long, similar to many Archean greenstone belts. It consists of a series of imbricated sheets of serpentinized ultramafic rocks, olivine gabbro, layered gabbro, chert, shale, siltstone and mélange [78].

The Erdene Uul ophiolite (Fig. 4D) is located ∼180 km SE of the Khantayshir, in the southern Lake Zone, and consists of isolated outcrops of serpentinized peridotites, cumulate gabbros and anorthosites, pillow lavas, and deformed agglomerates and tuffs [78], suggesting an arc-related origin. High pressure eclogites are present along the SE side of Erdene Uul, with Ar-Ar cooling ages of 540 Ma [81], possibly related to subduction in the Ediacaran-early Cambrian, similar in setting to the sub-Fleur de Lys eclogites of Newfoundland [82].

### Summary of the tectonic history and crustal growth of the Altaids

**Figure 5.**
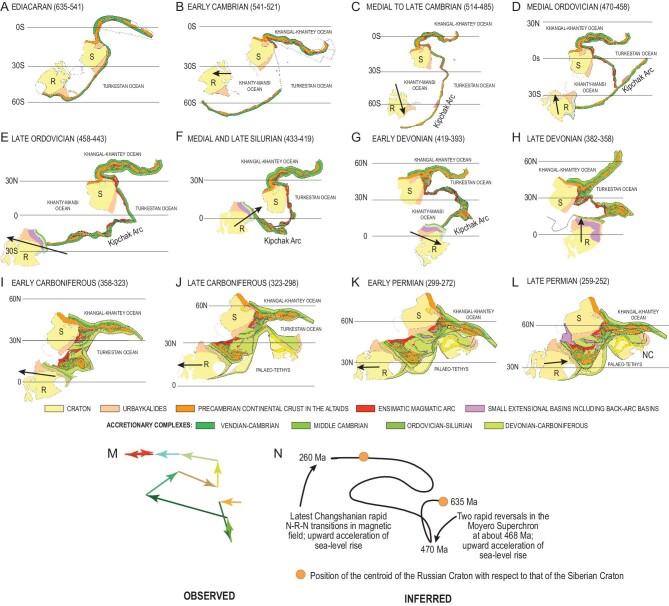
(A-N) Tectonic evolution of the Altaids (modified from Şengör *et al*. [1]).

Figure 5 illustrates the evolution of the Altaid superorogenic system after Şengör *et al*. [1] and the references cited above. The reason it is called a ‘superorogenic’ system is because it consists of three *independent but interrelated* orogenic belts, one along the Kipchak Arc and the others on both sides of the Tuva-Mongol continental ribbon.

During the Ediacaran, a long ensialic arc was rifting from the combined Russian and Siberian cratons, while two subduction zones were simultaneously active on both sides of the Tuva-Mongol continental ribbon, perhaps not entirely dissimilar to the present-day Philippines. By the early Cambrian, the rifted continental sliver had separated entirely from the Russian and Siberian cratons forming the backbone of the Kipchak magmatic arc, while the Russian and Siberian cratons were also separating from one another. The black arrows show the motion of the Russian Craton with respect to Siberia, and the latitudinal lines indicate the relative motion of the entire Altaid collage with respect to the magnetic poles (assuming a bipolar Earth). Notice that the Kipchak Arc was entirely detached from Siberia during the early Cambrian with only a long transform fault connecting the two. During the medial-to-late Cambrian this long transform fault had turned into an ensimatic island arc. This arc began sliding along a transform fault behind the Kipchak Arc left-laterally in the early and medial Ordovician, but the Kipchak Arc caught up with it by the end of the Ordovician, when the Russian craton whizzed westward with respect to the Siberian Craton. This motion was reversed during the Silurian, and the Russian Craton began to overtake the Siberian Craton eastwards during the early Devonian, accentuating the oroclinal bend of the Kipchak Arc that had formed during the Silurian. While that orocline tightened by almost a factor of two in the late Devonian, the Tuva-Mongol system slid southward creating the Altay orogenic collage with the Kuznetsk pull-apart basin.

In the early Carboniferous, a wide continental ‘bridge’, the ‘Kazakhstania’ of older literature(see review in Ref. [78]), had already been assembled and began growing rapidly towards the Turkestan Ocean by subduction-accretion. In the late Carboniferous, the Tarim Craton and the North China assembly hit and sealed off the Kazakhstan part of the Altaid collage. In the early Permian, only a small remnant of the Solonker Ocean was left between the Manchuride/North China assembly, which became totally obliterated by the late Permian. The closure of the Khangai-Khantey Ocean in Mongolia was an entirely Mesozoic affair lasting into the early Cretaceous (by hidden subduction after the late Triassic), thus ending the Altaid evolution.

Notice that during the early Carboniferous to late Permian interval, the relative motions of the Russian and the Siberian cratons had not ceased, although no oceans were left between them. In fact, the westerly motion of the Russian Craton between the early Carboniferous and the early Permian and its reversal in the late Permian created a wide belt of deformation across the western Altaids, creating the many intracontinental basins and eventually ending up in the ‘post-rifting’ bovine-head subsidence of the West Siberian Basin.

Figure 5M shows the relative motion path of the Russian and Siberian cratons that was involved in the Altaid evolution. As far as we know, this is the first time that a complete ‘displacement path’, during the evolution of such a vast orogenic system, has been attempted in pre-Jurassic evolution. Notice that the only abrupt change of course of the Russian Craton with respect to the Siberian Craton occurs during the medial Ordovician, when there were two rapid magnetic reversals of the magnetic field and an onset of sea-level rise. One wonders to what extent rapid reversals of convection in the outer core influence the convective circulation in the upper mantle.

All this evolution is extremely similar to what one sees in the Archean cratons of the world [83,84]. The Saharides of northern Africa and the Arabian Peninsula represent an orogenic collage of latest Precambrian age that is very similar to the Altaids both in overall structure and history of development [9,85]. Şengör and Natal’in [86,87] describe other examples of similar development both from the Phanerozoic (the Nipponides) and the Archean (the Yilgarn Craton). Below, we show that the Archean Superior Province of the North American Craton shows a high degree of similarity to the Altaids, in terms of its map patterns, rock types and structural/tectonic development.

### Structure and evolution of the Archean Superior Province

**Figure 6.**
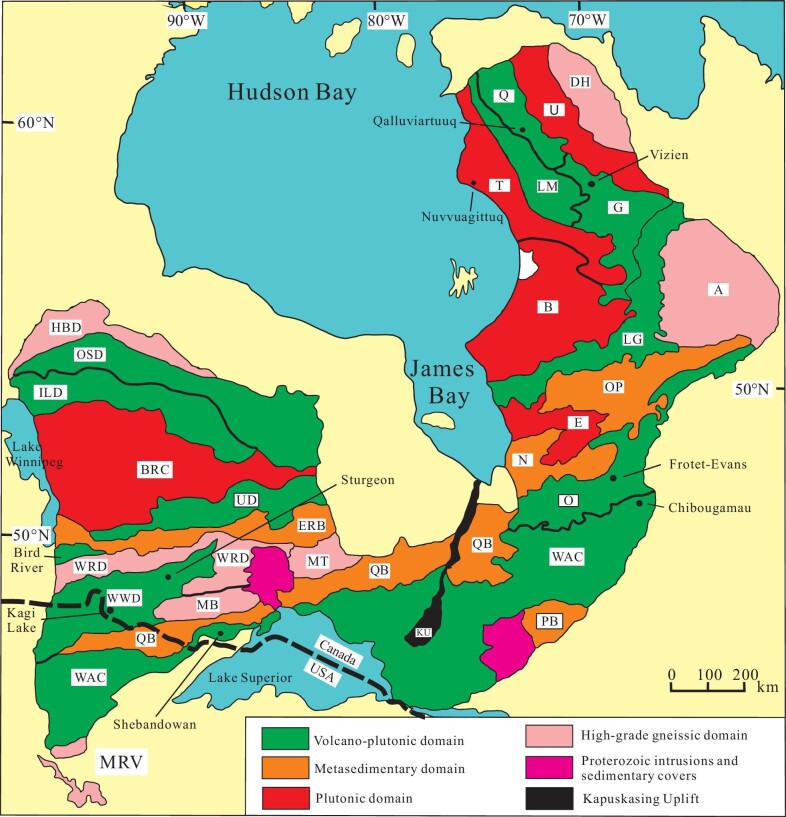
Simplified tectonic map of the Superior Province showing different tectonic belts and domains discussed in the text (modified from Percival *et al*. [2]). A, Ashuanipi; B, Bienville; BRC, Berens River Tonalite-Trondhjemite-Granodiorite (TTG) complex; DH, Douglas Harbour; E, Eastmain; ERB, English River Belt; G, Goudalie; HBD, Hudson Bay Domain; ILD, Island Lake Domain; KU, Kapuskasing Uplift; LG, La Grande; LM, Lac Minto; MRVT, Minnesota River Valley; MB, Marmion Block; O, Opatica; Op, Opinaca; OSD, Oxford Stull Domain; PB, Pontiac Belt; Q, Qalluviartuuq; QB, Quetico Belt; T, Tikkerutuk; U, Utsalik; UD, Uchi Domain; WAC, Wawa-Abitibi subduction/accretion complex; WRD, Winnipeg River Domain; WWD, Western Wabigoon Domain. Detailed descriptions of the various tectonic units in the map are provided in Supplementary Data.

The Superior Province of the North American Craton (*sensu* [84]) is the largest (1.4 × 10^6^ km^2^) intact portion of an Archean craton on Earth, and it is recognized as a collage of numerous amalgamated protocontinental and oceanic subduction/accretionary complexes and arcs that extend for over 2000 km along the strike (Fig. 6). These have been named terranes in the Canadian literature, although, where possible, we refer to the different units simply as tectonic subprovinces, units or domains, or abandon the groupings into terranes completely. This is necessary as some terranes are defined based on their ages, others on their lithological components and still others on their metamorphic grade. In some cases, ‘terrane boundaries’ (such as that of the Riviere Arnaud) even cut across different tectonic domain or subdomain boundaries. Application of the terrane terminology has, in our opinion, hindered tectonic understanding of the Superior Province, and the following represents our first attempt to correct this trend [39]. In the following, we analyze the geology using structure, ages, rock types, chemistry and metamorphism to help decipher the tectonic evolution of the province, with detailed descriptions included in Supplementary Data. We pay particular attention to the character of ophirags, which in the Superior are generally interpretable as being from the bases of arcs, backarc basins, forearc basement, MORBs in accretionary wedges, and fragments of oceanic plateau. We end with a discussion and comparative orotomy (*sensu* [1,88]) of the Superior Province and the Altaids.

**Figure 7.**
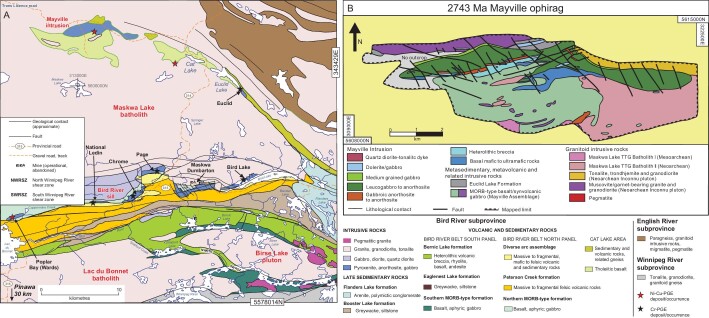
(A) Map of the Maskawa Lake batholith and the southern arm of the Bird River greenstone belt, with the locations of the Bird River sill, and the 2743 Ma Mayville ophirag (modified after Ref. [97]). (B) Detailed map of the Mayville intrusion (map from Ref. [93] after Ref. [97]). See Supplementary Data for more information.

Previous work (summarized by Percival *et al*. [2]) led to the recognition of five main orogenic events that ended double-sided accretionary tectonic and magmatic intervals, generating extensive flysch sequences and crustal-scale imbricate structures. The Superior orogenic system includes old fragments with Eoarchean to Mesoarchean ages and long structural histories (Fig. 6), which were assembled by the closure of Neoarchean oceanic tracts between 2720 and 2680 Ma that formed the main E–W strike of the belts within the craton, and generated large strike-slip fault systems between some of the accreted tectonic units [2]. Most of the Neoarchean magmatic rocks have arc-affinities generated by subduction of Neoarchean oceanic lithosphere, giant slabs of which are still preserved in some places and imaged geophysically to the offset Moho and, based on magnetotelluric data, penetrate perhaps as deep as 300 km [89,90]. Ophiolites and ophirags have been described (e.g. [44,91–93]) but most of the mafic rocks in the Superior Province are interpreted to be fragments of arcs [94]. Most of the well-studied ophiolitic fragments and ophirags of the Superior Province are identified as preserved remnants of the simatic bases of arcs—upon which younger volcanics were extruded and arc-related plutonic suites intruded—and are best preserved where the older ophiolitic basement is transitional in character between the arcs and backarcs or forearcs (e.g. Bird River and Mayville ophirags, Fig. 7). Other ophiolitic fragments have been interpreted as accreted oceanic plateau, and a few ophirags with MORB characteristics have been described from some of the accretionary, dominantly metasedimentary terranes.

The broad E–W strike of the different accreted units in the south is deflected to the N–NW as the belts continue into northern Quebec (Fig. 6), where they were strongly reworked and metamorphosed at circa 2730–2680 Ma. The change in orientation (oroclinal bend) of accreted oceanic, arc and continental units is remarkably similar in scale and component characteristics to similar areas of the Altaids orogenic system (Fig. 1), which is the world's largest Phanerozoic Turkic-type orogen [1]. Late tectonic events include large-scale strike-slip faulting with associated deposition of the ‘Timiskaming’ conglomerates, alkaline magmatism, and granite intrusion with associated gold mineralization.

There is debate about the significance of the high-grade Kapuskasing Uplift (Fig. 6), but geologic and paleomagnetic data [95,96] are consistent with the zone accommodating a 14°CCW rotation about a pole located at 51°N 85°°W, related to Trans-Hudson orogenesis [3].

 

### Discussion: can comparative orotomy yield insights into the mechanisms of continent formation?

Based on the geological characteristics of different subprovinces in the Superior Province (see Supplementary Data), and reviews by John Percival, Kent Card and co-workers [2,98] and numerous reports before and after those two seminal papers, it is recognized that the Superior Province consists of a series of different tectonically juxtaposed subduction/accretionary complexes, intruded by migrating arc fronts, then involved in continental-margin accretion, additional arc magmatism and collisions with other arcs, oceanic plateaus and ribbon continents. Our review shows that the individual tectonic belts and their contained ophirags formed in different tectonic settings and show different stages in the formation and evolution of continental crust [6,7,44,56,83]. Some of the ophirags in the subduction/accretion complexes are clearly remnants of former oceans, with the largest concentration in the Wawa-Abitibi subduction/accretionary complex (Fig. 6). Some of the belts include juvenile arcs, accretionary wedge complexes and accretionary wedges that the arc magmatic fronts migrated through, and some are continental arcs. These have experienced several different convergence events that led to the formation of protocontinental blocks that then collided, generating late-stage granitoids, and began to form the ‘greater Superior Craton’ (as some parts have since been rifted away) as one of the earliest (preserved) sections of a large emergent continent on the planet, which in some speculations may have been part of an even larger first supercontinent, appropriately named Kenorland [99–101]. The ancient Superior Craton experienced several rifting and re-amalgamation events along its borders, and now is a smaller (but still the world's largest surviving) fragment of an Archean craton, preserved as the Superior Province within the North American Craton [6].

The multiple types of similarities between the Altaids and the Superior Province are clear from the maps and descriptions, and from the discussion above and in Supplementary Information 1 and Figs S1–S2. The most striking are: (i) the overall size and types of different tectonic units that comprise both orogenic systems. (ii) The craton-scale distribution of these different tectonic units, into a broad 90° oroclinal bend, bounded by younger external orogens. (iii) The presence of internal ribbon microcontinents with 180° oroclinal bends, and displaced fragments, bounded by strike-slip fault systems. (iv) The sequence of development from early oceanic accretion and formation of intraoceanic arc systems with their associated subduction/accretionary wedges, through which arc magmatic fronts migrated. Continued convergence led to collisions that formed continental-margin arcs, then eventually larger protocontinents, followed by eventual continent–continent collision, thickening the orogen. These early events were followed by (v): generally dextral strike-slip translations along arc/forearc/microcontinental boundaries, which continued from early subduction to arc–arc collisions, then to arc–continent collisions, resulting in the different belts, with generally similar ages, being stacked into a nearly equant craton with dimensions remarkably similar to the Altaid orogenic collage (Fig. 1).

Below, using the lessons from the Altaids, we take the first steps towards a better understanding of the formation of Archean cratons. We make several recommendations, the most important of which are: (i) abandon the terrane terminology, and instead search for classic orogenic zonations, and understand the origins and histories of different tectonic belts instead of classifying them into different types that tell us little about their history; (ii) the role of strike-slip faulting is now well understood in the Altaids (e.g. [69,71,72]) and is readily apparent from the map patterns of the Superior Province, but the significance of this faulting is greatly underappreciated. Both orogens evolved throughout much of their history with oblique (mostly dextral in both cases) convergence and post-collisional displacements. In the Altaids, these displacements, and the resulting stacking of originally continuous arc belts into an equant orogenic collage, is well established [69,71]. In the Superior Province, most geologists acknowledge the long-term dextral transpressional deformation, yet with a few exceptions [102–104] do little to incorporate possible huge strike-slip translations and repetition of formerly continuous arcs into a map-view duplex with an oroclinally folded (by strike-slip deformation) ribbon continent in its core, as in the Altaids (Fig. 1).

Thus, in the sections below, we review the tectonic development of the Superior Province, applying at will lessons from the Altaids, to spur further research into the development of the Superior Province and cratons in general, and to better explore similarities and differences with Phanerozoic areas of massive crustal growth.

### Old ribbon protocontinents with rift sequences

**Figure 8.**
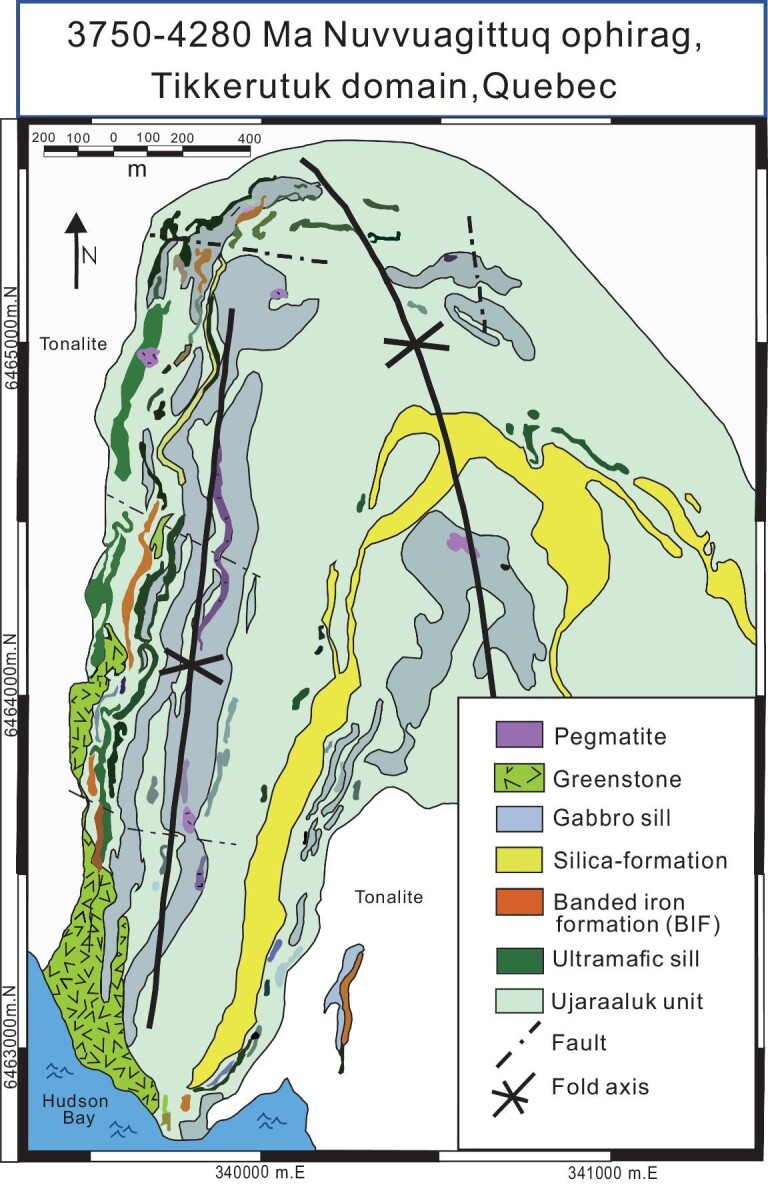
Simplified geological map of the Eoarchean Nuvvuagittuq belt of the northeastern Superior Craton outlining the distribution of pillow basalts, bedded cherts, banded iron formations and intrusive sills. Modified after Ref. [106]. The Ujaraaluk unit is an amphibolite-facies metavolcanic unit consisting mostly of basalt to basaltic andesite. See Supplementary Data for additional description.

**Figure 9.**
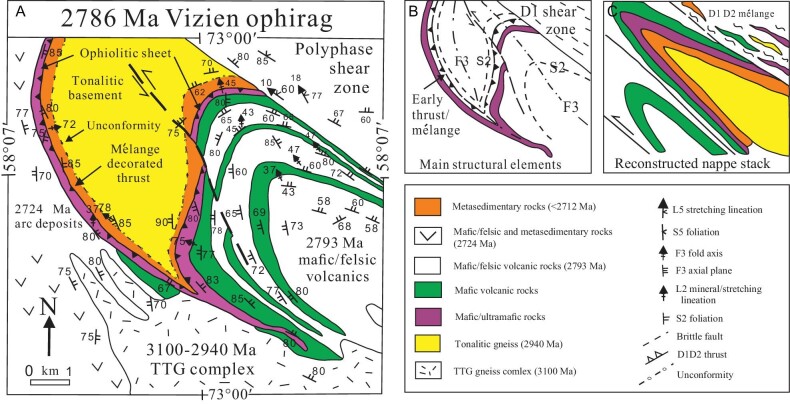
(A) Structural map of the Vizien greenstone belt (drawn after Refs [108–111]). Mafic/ultramafic rocks of the Vizien ophirag were thrust over circa 2940 Ma tonalitic basement at circa <2712 Ma, generating a thin mélange zone, then folded several times and emplaced over an older circa 3100 Ma TTG terrane hosting a 2724 Ma arc sequence. Note that F4 structures are not shown on the map, as they do not affect the map pattern. (B) Main structural elements of the map, drawn after Refs [108,109]. (C) Schematic reconstruction removing F3 folds, showing earlier structural arrangement of the ophiolitic rocks thrust over the tonalite, and folded into a fold/thrust nappe stack with a structural style similar to that of accretionary wedges. See Supplementary Data for additional description of the map.

Some of the belts of the Superior Province represent older protocontinental fragments with pre-Neoarchean histories, which saw continental-margin subduction-accretion complexes built on their edges, and which later collided to form larger protocontinents. The Hudson Bay domain occupying the northern part of the craton, if the correlation [2] with the Tikkerutuk, Bienville, Lac Minto and Goudalie domains is correct, would be the largest ancient protocontinent (Hudson Bay block) in the Superior Craton, and has rocks as old as Eoarchean and Mesoarchean in age but was strongly affected in places by Neoarchean magmatism and deformation. Interestingly, the oldest rocks in the Tikkerutuk domain (and among the oldest on Earth) at Nuvvuagittuq (Fig. 6) preserve fragments of oceanic crust and overlying hydrothermal, fossiliferous deposits and associated deep-sea sediments [105–107] encapsulated in a sea of circa 3.66–3.8 Ga subduction-related Tonalite-Trondhjemite-Granodiorite (TTG) and more felsic intrusions, making Nuvvuagittuq the world's oldest ophirag (Fig. 8). Other ophirags in the Goudalie, Qalluviartuuq and Lac Minto domains are younger [108–111], consisting of altered peridotites, gabbros and basalts, and reflect subduction-related accretion of circa 2.790 Ma oceanic crust (normal or thickened crust in an oceanic plateau) at locations including Vizien (Fig. 9) in the Goudalie domain northeast of Hudson Bay.

In the Neoarchean at circa 2720 Ma the Hudson Bay block NW of Hudson Bay collided obliquely with the Oxford Stull and Island Lake accretionary complexes bordering the Berens River TTG-dominated arc system, forming zones of dextral shearing and greenschist facies metamorphism along its southern margin [107]. The nature of the Hudson Bay block remains obscure. In the west it consists of arc-type plutons active from 3.2–3.1, 2.85–2.81 and 2.74–2.71 Ga, suggesting a continental-margin arc affinity. In the east, however, older rafts of ophirags with ages up to ∼4.0 Ga, engulfed in similar-aged arc plutons (e.g. Nuvvuagittuq, Fig. 8), led Percival *et al*. [2] to correlate the different tectonic belts (using a terrane analysis approach) on both the eastern and western sides of Hudson Bay into one domain, or block, that they considered continental in aspect at the time of its collision with the Berens River arc at 2720 Ma. We note that arc magmatism was continuing through the time of the collision, so suggest alternatively that the Hudson Bay block was a continental-margin arc system, with older relicts of its former history embedded in a sea of arc plutons, as is common in continental-margin arc systems [8].

The 2720 Ma strike-slip systems related to the collision of the Hudson Bay block and Berens River arc are superimposed on the earlier accretionary complex of >2.73 Ga ophirags imbricated with 2.88–2.73 juvenile and continental-margin arc suites in the Oxford Stull domain that forms the subduction-accretion complex along the north margin of the Berens River arc (Fig. 6). The Berens River arc system also contains numerous Mesoarchean remnants, but was widely intruded by the 2745–2695 Ma Berens River plutonic suite and shows very little deformation related to Neoarchean collision [107]. At Lake Winnipeg and Caribou Lake in the Berens River arc (Fig. 6), a sequence of quartzite-komatiite-banded iron formations, cut by circa 2920 tonalitic dikes, are interpreted as rift sequences [104,112,113].

The Winnipeg River ribbon microcontinent (Fig. 6) also contains old, circa 3.4 Ga crustal remnants, but was multiply deformed and intruded by numerous suites of plutons during Neoarchean (2710–2690 Ma) tectonism [114]. Structures in the Winnipeg River ribbon microcontinent have an interesting history, as early field geologists considered the map patterns to result from diapiric and vertical tectonic processes, whereas later detailed structural analyses [115–118] have shown that they result from a combination of early thrusting and folding, followed by late ductile transcurrent shearing. A similar scenario has recently been proposed for the Pilbara Craton, where dome-and-basin structures have for some years been thought to indicate that the Mesoarchean was dominated by vertical drip and antidrip tectonics, but were then shown by T Kusky *et al*. [7] to be nothing more than vertically intruding and re-folded plutons in the root of an accretionary-to-continental-margin arc system, much like the Altaids [7,8]. The Winnipeg River ribbon microcontinent is apparently oroclinally folded into an isocline (Fig. 6), very similar in style and size to the Central Mongolian (also called the Tuva-Mongol ribbon continent, as in Fig. 3) microcontinent of the Altaids (Figs 1 and 2). The Marmion block on the southern edge of the Winnipeg River ribbon microcontinent is probably just a sliver of the microcontinent, sliced off by late dextral strike-slip faulting, as suggested by Kusky and Hudleston [102], and similar to blocks displaced from the Tuva-Mongol (or Central Mongolia Microcontinent) ribbon continent, as described by Khukhuudei *et al*. [78].

**Figure 10.**
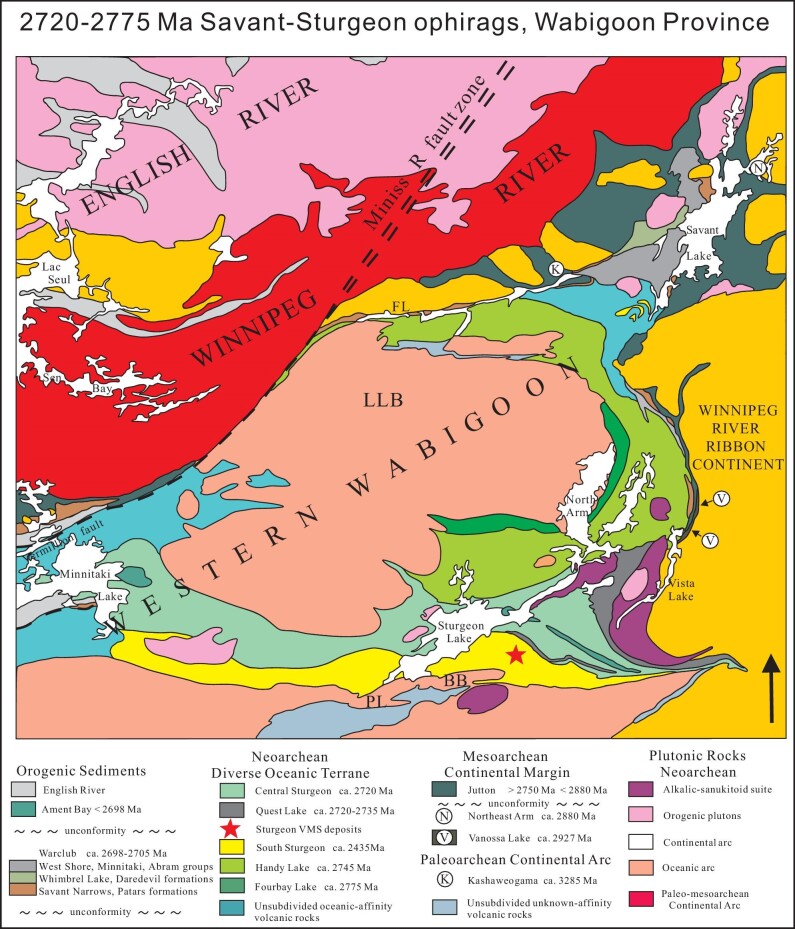
Map of the Savant-Sturgeon greenstone belt (modified after Sanborne-Barrie and Skulski [118]), showing volcanic, sedimentary and plutonic assemblages, including the Savant-Sturgeon ophirags located along the suture between the Winnipeg River ribbon continent and the Western Wabigoon subduction/accretionary/arc complex (see location in Fig. 6). Note the abundant late strike-slip faults. Abbreviations are for pluton names, including FL-Fairchild Lake intrusion, LLB-Lewis Lake batholith, BB-Breidelman Bay pluton and PL-Pike Lake gabbro.

Remnants of rift-deposits resting unconformably on these old protocontinental fragments are locally preserved [119], as they are in the Slave Province [52,120]. These typically include basal quartz- or gneiss-pebble conglomerates, sandstones, with or without mafic/ultramafic lavas, and rarely, as at Steep Rock Lake (Fig. S1) in the Marmion block, a thick carbonate sequence [102]. Similarly, in the Winnipeg River ribbon microcontinent, komatiites at Lumby Lake (2.83 Ga, [121]) and a sequence of basal clastics overlain by 5 km of continental tholeiites at Sturgeon-Savant Lake (Fig. 10) have also been interpreted as a >2750 Ma plume-induced rift deposit [122], although the volcanic sequence is more likely allochthonous as described in Supplementary Data 1. Undoubtedly, the best rift-passive margin sequence is exposed at Steep Rock Lake where a <2780 Ma clastic-thick carbonate sequence overlies the circa 3.09 Ga Marmion gneiss [102]. In the northern La Grande domain, ancient 3.33–2.79 Ga gneisses are overlain unconformably by <2750 Ma quartz arenites and 2820–2730 Ma komatiites and basalt [123].

The Opatica domain has late Mesoarchean circa 2820 tonalitic gneisses, with 2770–2700 arc and backarc assemblages, intruded by late-post-tectonic granitoids, and may be a slightly older arc system adjacent to which the Wawa-Abitibi oceanic accretionary complex was built [44,124]. Finally, rocks of the Minnesota River Valley Gneiss Complex have remnants of very old crust preserved as remnants or enclaves in 2680–2650 Ma granitoids, and may represent the southern continent of the Superior Craton.

### Ophirag-bearing subduction-accretion complexes, with migrating arc fronts

Vestiges of early oceans, including ophiolites, ophirags and overlying OPS sequences, are preserved in some of the domains of the Superior Craton, in belts that we regard as preserved segments of Archean subduction-accretion prisms of these orogens (*sensu* Kusky and Polat, [44]). The Wawa-Abitibi belt contains the largest assemblage of ocean-derived rocks in the Superior Craton, extending approximately 1200 km with a width of 200 km (Fig. 6). Numerous studies [2,125] have recognized at least eight circa 2790–2697 Ma submarine volcanic complexes including komatiites, and tholeiitic to calc-alkaline volcanics. The tectonic setting of the Abitibi belt in particular is hotly debated and a focus of current extensive research (John Percival, personal communication, 2022), with some geologists favoring arc and oceanic imbrication [126,127], others favoring a plume-arc interaction [128] and still others proposing a non-plate tectonic stagnant lid/plateau dynamic regime unlike anything on modern Earth [129]. Canadian LITHOPROBE studies show consistent north-dips, and the continuation of greenstone belts to an offset Moho, consistent with south-directed thrusting of the assemblage [89,90]. This suggests that the belt includes rather large allochthonous thrust sheets emplaced towards the south, without intense internal thrust imbrication. The volcanics are unconformably overlain by the circa 2690 Ma sedimentary sequence of the Porcupine Group [130]. The belt is intruded by 2690 Ma batholith complexes that were emplaced at depth concurrently with volcanism, some of which have been folded to expose mid-crustal levels (∼700 MPa) [131].

The Western Wabigoon domain (Fig. 6) consists of an assemblage of sinuous metavolcanics/plutonic and sedimentary greenstone belts, surrounding domical granite batholiths [132]. The oldest rocks in the greenstone belts consist of juvenile submarine tholeiitic basalts, and a 2775–2720 Ma calc-alkaline suite of basalt-andesite-dacite, and these are all intruded by juvenile tonalitic plutons that were emplaced between 2735–2730 Ma [133,134]. This sequence was suggested to represent a backarc sequence built on an ophiolitic or oceanic plateau basement (e.g. the Savant-Sturgeon Lake ophirags, Fig. 10), and overlying 2710–2700 Ma clastic sediments have an age spectrum that suggests erosion from the Western Wabigoon domain and the Winnipeg River ribbon microcontinent during their collision during the central Superior Orogeny [2,118].

The Oxford Stull and Island Lake domains contain sheets of oceanic-affinity juvenile submarine tholeiitic basalts imbricated along 2820–2780 Ma D_1_ thrust faults with continental-margin calc-alkaline mafic-felsic volcanics and associated clastic sediments [135]. In the western Uchi domain, a group of 2723 Ma tholeiitic basalts and calc-alkaline andesites in the Black Island assemblage are interpreted by Bailes and Percival [136] as an oceanic backarc assemblage, formed during the closure of the ocean between the Winnipeg River ribbon microcontinent and Berens River arc with its added accretionary complexes. The associated arc built on the western Uchi domain is called the Bidou assemblage, and includes 2723–2718 Ma tholeiitic basalt overlain by calc-alkaline basalt and dacite [137].

In the east-central English River belt, the Melchett Lake greenstone belt consists of calc-alkaline dacite with juvenile Hf isotopic signatures, and coarse clastic sedimentary rocks that Davis *et al*. [138] have identified as a remnant of an intraoceanic arc complex. In the Qalluviartuuq and Goudalie domains of the NE Superior Province, greenstone belts such as Qalluviartuuq and Payne Lake include strongly deformed sequences of 2840–2830 Ma juvenile tholeiitic and calc-alkaline basalts and andesites, identified by Skulski *et al*. [139] as remnants of an oceanic plateau (or just thick Archean oceanic crust; c.f. [48,140]), with similar sequences in the Buet, Pelican, Nantais and Duquet greenstone belts. In contrast, in the Vizien greenstone belt of the Goudalie domain, a tectonic slice of 2786 Ma peridotite-basalt of oceanic plateau affinity structurally overlies a continental clastic sequence, forming a basal mélange and depositing thin associated clastic sediments during emplacement (Fig. 9).

### Continental-margin magmatic arcs

Figure 6 shows that large portions of the Superior Province are formed of plutonic/volcanic complexes that are generally calc-alkaline TTG-type intrusive suites and their extrusive equivalents, whereas other areas—especially units in the NE part of the province—have nearly exclusively plutonic assemblages that extend for hundreds of kilometers [2], similar to those of the Altaids described above. The Oxford Stull and Island Lake subduction/accretion/arc systems located north of the Berens River arc host six suites of circa 2860–2710 Ma plutonic rocks [107] that include tonalites, hornblende-bearing tonalites, biotite granites and a younger (2710 Ma) sanukitoid suite comprised of peraluminous granites and diorite-monzonite. Isotopic signatures of the older suites show components from both the mantle and partial melting of older crust, whereas the sanukitoids appear to be derived from metasomatized mantle [139]. They suggest that the older calc-alkaline magmas represent proto-continental-margin arc volcanism during subduction under the Berens River arc, and that ocean closure and collision with the Hudson Bay northern continent at 2720 Ma terminated the arc magmatism, but was followed by slab break-off magmatism, as shown in the youngest orogens such as Papua/New Guinea (e.g. [141]). The south-central part of the Berens River arc hosts an additional continental-margin arc, preserved as the 2745–2708 Ma Berens River plutonic complex [142]. As in the arc on the northern accreted belts of the Oxford Stull and Island Lake domains, the Berens River arc magmatism is followed by a suite of 2700–2698 Ma slab-break-off-related magmatic rocks.

A Java-style arc built on the Winnipeg River ribbon microcontinent (Fig. 6) includes circa 2770–2680 Ma plutonic sheets interleaved with volcano-sedimentary sequences, and intruded by younger plutons [143,144]. The 2725–2705 Ma calc-alkaline TTG suites show mixed mantle and assimilated crustal sources [145], but this phase of magmatism ended at 2700 Ma and was followed by adakitic magmatism at 2702 Ma along with deformation and sedimentation [146], then experienced mantle-derived sanukitoid mafic magmatism. Younger leucogranites formed during crustal thickening and melting [113]. The arc on the Winnipeg River microcontinent is interpreted to have formed from north-directed subduction of rocks preserved now in the subduction/accretionary belts in the Western Wabigoon and Abitibi oceanic belts, from at least 2750–2705 Ma, followed by closure of the Wawa-Abitibi ocean and collision with the Minnesota River continent on the opposite side of the Wawa-Abitibi ocean. This was succeeded by slab-break-off-related sanukitoid magmatism [2,133].

The NE Superior Province (Fig. 6) hosts a late Archean continental-margin-style plutonic belt (e.g. [8]), with several suites of 2760–2710 Ma low-K TTG, 2730–2680 Ma hornblende-quartz monzodiorite and granodiorites and granite, 2730–2710 Ma pyroxene granites, and 2720–2700 Ma mafic-ultramafic plutons [147,148]. These plutonic belts cut across the boundaries of the older subduction/accretion belts, and have been identified as a continental-margin magmatic arc suite generated by N-dipping subduction [149] followed by younger melts formed during collision. Much of the NE part of the shield was affected by magmatism over wide areas from 2790–2680 Ma, with vertically walled plutons with N–NW strikes composing most of the bedrock [132,148,150]. The oldest of these (2790–2760 Ma) is a juvenile low-K TTG suite that shows some inheritance of older (circa 2.9 Ga) crust [151,152]. A similar suite of low-K TTG intruded from 2760–2710 Ma and shows contamination of 3.9–3.0 Ga crust [153]. The interval from 2740–2680 Ma saw intrusion of pyroxene granites and granodiorite-granite suites, and then a 2715–2680 Ma suite of diatexites, granodiorites and granites related to crustal melting following continent–continent collision [111].

### Colliding arcs and micro-continents: sutures of the Superior Province

To understand the sequence of collisions of juvenile arcs, continental arcs and plateaus, and the eventual collision of protocontinental blocks to form the ancestral ‘greater Superior Craton’, Percival *et al*. [2] synthesized data on the nature and timing of magmatism, sedimentation, deformation and metamorphism in the various belts of the Superior Province, then identified the types and ages of sutures that bound the different belts. They indicate that the isolated older crustal fragments, including the Hudson Bay domain, formed a larger continent by 2715 Ma, and that the Oxford Stull and Island Lake subduction/accretionary belts were attached to the Berens River arc. This arc and hypothesized continent are suggested to have collided at 2715 Ma by the change in provenance of detrital zircons at that time [139], with the intervening ocean closing by subduction beneath the Oxford Stull domain, which incorporated slivers of oceanic lithosphere along the continental-margin magmatic arc [107]. While generally <500 km wide, this amalgamated arc apparently served as the upper plate in continental-margin-style subduction systems (*sensu* Kusky and Wang [8]) for more than 200 Ma, with magmatism from 2940–2710 Ma, ceasing during a major deformation event at 2720 Ma, which led to the deposition of clastics including syn-orogenic flysch that filled the English River basin at 2700 Ma, covering the suture between the arc and Winnipeg River microcontinent [104].

A north-dipping seismic reflector [90] coupled with surface geology shows that the boundary between the Wawa-Abitibi subduction/accretion oceanic assemblage and the Winnipeg River micro-continent (with the accreted Western Wabigoon subduction/accretionary complex) is a north-dipping suture marked by greenstone belt ophirags that, followed downwards, offset the Moho along the extension of the surface structure (with strong shear-wave anisotropy) (Fig. 6). These are best interpreted as the remnant of a thick slab of oceanic crust (ophiolite) outlining the trace of an Archean paleo-suture [103,153]. Magmatism on the upper (Wabigoon-Winnipeg-River continent) plate ended at 2703 Ma, and was followed soon afterwards by deformation at 2700–2695 Ma along the 50–100 km wide Wawa-Abitibi/Wabigoon suture, depositing flysch into the Quetico basin [154,155].

The southern boundary of the Wawa-Abitibi oceanic complex is sutured against the Minnesota River Valley microcontinent in the west and Pontiac subprovince in the east, manifested as a north-dipping seismic reflector [90]. The northern boundary of the Abitibi belt in the eastern Superior is also sutured against the Opatica belt to the north, also associated with a north-dipping 2690–2685 Ma paleo-subduction zone at depth [156,157]. The suture zone is characterized by thrust and strike-slip faults intruded by many late plutons on the surface [158,159].

In the NE Superior Province, the ancient rocks (3.9–2.9 Ga) in the Tikkerutuk and Bienville domains are apparently sutured against the Utsalic and Douglas Harbour domains to the NE, along a strongly deformed belt of oceanic-affinity greenstones that runs through the Goudalie domain [111], where the 2786 Ma Vizien greenstone belt (Fig. 9) is thrust over 2940 Ma basement and its <2718 Ma autochthonous conglomeratic cover [110]. Suturing was followed by granulite facies metamorphism and partial melting from 2710–2690 Ma [160].

### Comparative orotomy of the Archean Superior and Phanerozoic Altaid orogenic systems

The collision of the various arcs with associated subduction accretion complexes and older proto-microcontinents of the Superior Province in the late Archean formed one of Earth's earliest continents, which may have been a part of an even larger early supercontinent called Kenorland [99–101] after the Kenoran orogeny (named in turn after Portage-aux-Rats, or Kenora near Lake of the Woods in Ontario) [161–163]. Sutures are generally marked at present as wide zones of ductile transpressive dextral shear [164–168], much as in the Altaids [1], with some zones showing associated magmatism, sedimentation and metamorphism [2,35,169]. Some of the oldest accretionary phase history of the Archean Superior orogenic system is presently obscure and undoubtedly involved accretion of many slices of oceanic lithosphere (some of which were illustrated above and in Supplementary Data) and oceanic sediments, forming mélanges and thrust belts (e.g. [44]). In this section, we focus on the late events, which amalgamated the already formed protocontinents, arcs and oceanic domains.

The circa 2720 Ma Northern Superior Orogeny is related to the collision between the presumed ancient continent of the Hudson Bay domain and Berens River arc, recorded in the Oxford Stull and Island Lake oceanic accretionary complexes [139]. Subduction polarity was to the south, forming the continental-margin-style arc [8] on the accreted Oxford Stull and Island Lake complex and the north margin of Berens River arc, and also shown by the north-vergent thrusting along the boundary [164]. This is supported by a zone of high resistivity and seismic velocity that dips under the North Caribou unit from the boundary, which is likely the remnants of the closed oceanic lithosphere preserved to ∼150 km depth [90]. The postulated northern Hudson Bay continent and the Berens River arc were isolated until 2715 Ma, when they suffered a common deformation/metamorphic event related to their collision, deposition of the <2704 Ma Cross Lake synorogenic sedimentary wedge [165], and eruption of circa 2710 Ma shoshonitic volcanics in extensional jogs in dextral strike-slip systems [135] related to oblique subduction slicing the overlying arc, as in Sumatra and the Altaids [1,69,153]. The Bird River greenstone belt includes the Bird River and Mayville ophirags (Fig. 7), which are trapped along this suture.

The 2720–2700 Ma Uchian Orogeny represents the continent–continent collision of the Winnipeg River ribbon microcontinent and the Berens River arc through closure of an ocean basin through north-directed subduction beneath the Uchi accretionary complex on the southern side of the arc [166]. The suture is covered by <2705 Ma sediments of the English River basin, cut by late dextral faults [167], again showing long-term oblique subduction and slicing of the upper plate arc system, as in the Altaids [1,70,71]. Circa 2718–2712 Ma, structures associated with the collision dipped steeply N, and were intruded by 2700 Ma plutons [142]. Next, the Berens River arc and all of its accreted orogenic material on its southern and north margins were thrust to the south over the edge of the English River basin.

The 2700 Ma Central Superior Orogeny amalgamated several arcs and continental-composition slivers to form the first continent-sized mass of the proto-Superior Craton [2]. This early continent included oceanic lithosphere of the Western Wabigoon domain [122], accreted against the Winnipeg River/Marmion ribbon microcontinent. The Shebandowian Orogeny occurred at 2690 Ma, juxtaposing the Wawa-Abitibi oceanic complex with the growing protocontinent, through northward subduction associated with magmatic cessation at 2695 Ma in the amalgamated Wabigoon-Winnipeg River ribbon continent, and intrusion of slab-break-off-related sanukitoids at 2695–2685 Ma [133]. This was associated with rapid sedimentation in the Quetico basin from 2698–<2690 Ma [168], marking its transition from an accretionary wedge to a foreland basin [169]. The Wawa-Abitibi accretion to the Wabigoon-Winnipeg-Marmion ribbon continent at 2685–2680 Ma was associated with transgressive sedimentation and dextral transpression along the boundary [170], showing that the *Altaid-style* [1] strike-slip slicing of the accreted arcs and subduction/accretion complexes continued throughout the duration of their collision. Intrusion of Alaskan-type ultramafics and alkaline magmatism is interpreted to be related to slab break-off of the Wawa-Abitibi slab [114]. Seismic reflection profiles [90] show north-dipping reflectors, but the Quitico belt appears to be shallow and preserves the geometry of an accretionary prism.

### Terminal continent–continent collision

The 2680 Ma Minnesotan Orogeny represents the final continent–continent collision that amalgamated the Minnesota River Valley microcontinent with the rest of the ancestral Superior Craton, with the circa <2682 Ma Pontiac belt wedged in between, in the eastern part of the collision zone (Fig. 6). Seismic reflection suggests northward-dipping subduction beneath the Wawa-Abitibi belt, with the unexposed Great Lakes Tectonic Zone representing the terminal suture. This collision caused widespread deformation in the Wawa-Abitibi belt, Pontiac and Minnesota River Valley domains. In seismic refraction and reflection profiles [89,90], the Minnesota River Valley continent is the lower plate of the orogen, and projects downwards into a zone of high velocity interpreted to be a slab of oceanic lithosphere that was subcreted to the base of the crust at 2680 Ma [116].

Much of the history of the NE Superior Province is obscured by voluminous plutonism and high-grade metamorphism, but the Utsalik and Douglas Harbor arc systems appear to have collided with the ancient Hudson Bay northern continent at 2700 Ma, along the Qalliarvartuuq and Goudalie accretionary domains, causing high-grade metamorphism throughout the region [144]. Tracts of <2748–>2735 Ma metasedimentary rock, greenstone belts and Banded Iron Formation (BIF) in the Lac Minto, Goudalie and Qalliarvartuuq domains may represent tracts of the ocean that closed, and thus represent the terminal collision in the NE Superior Province.

### Late effects of continent–continent collision

Post-amalgamation events in the Superior Province continued for some tens of millions of years after the various arcs and microcontinents collided, and after the terminal collision of the Minnesotan Orogeny, forming one of the first truly large cratons on Earth (albeit later than the smaller Kaapvaal Craton, see Ref. [7]). The southern and western parts of the craton were affected by post-amalgamation sanukitoid intrusions, widely attributed to slab-break-off magmatism [114,171]. Other post-magmatic mantle-derived igneous rocks include the Shebandowan shoshonites [172,173], the Timiskaming volcanics [173], lamprophyres, nepheline syenites and carbonatites [174,175]. The most common post-amalgamation event was the intrusion of widespread, crustally derived granites and pegmatites between 2670 and 2620 Ma.

Concurrently with intrusion of the post-tectonic granites, the granulite-facies terranes were experiencing high-grade metamorphism and migmitization, ranging from 2690 Ma in the Pikwitonei domain [176], 2685–2665 Ma in the English River belt and 2695–2620 in the Winnipeg River subprovince [177]. Likewise, the discordant deep-crustal rocks of the Kapuskasing structure show high-grade metamorphism at 2680 and 2602 Ma [178]. In the east, the Ashuanipi complex saw high-grade metamorphism associated with migmitization and melt generation at 2680–2640 Ma [179]. Together, the high-temperature conditions of these gneiss terranes show that temperatures remained elevated at depth throughout the craton for more than 60 Ma after the main terminal collision ended in the Minnesotan Orogeny. In many places, these elevated temperatures are thought to be responsible for the release of late hydrothermal fluids that are associated with some gold deposits [180,181], many of which have ages of 2710–2610 Ma. Many of these deposits and other evidence for late-stage fluid release are associated with late-stage steep (generally transcurrent) faults [182], and may also have played a role in the fluid release and cooling of the deep crust, leading to ‘cratonization’, in a manner similar to that hypothesized for the Slave, Zimbabwe and other cratons [47,48,52]. Importantly, these long-lived *Altaid-style* [65,66] transcurrent fault systems likely repeat and stack once-continuous arc belts, forming the equant pattern of arcs and microcontinents with broadly similar ages across much of the Superior Province (Fig. 6), building a continent in a style remarkably similar to that outlined for the Altaids above, and by [1,69–71].

While the deep crust exposed in the high-grade gneiss terranes was experiencing HT metamorphism, many areas at the surface, especially in the south in the Abitibi belt, saw the deposition of the Timiskaming sedimentary sequences unconformably on the older basement between 2680 and 2673 Ma [183]. These were in most places associated again with strike-slip faulting, which is at present still underappreciated in the Superior Province. We suggest that by analogy with the Altaids, the role of strike-slip slicing and repetition of arc sequences needs to be more closely evaluated, to better understand the paleogeographic development of Earth's largest preserved Archean continental fragment.

## FUTURE RESEARCH IN COMPARATIVE OROTOMY

In this work we have applied the principles of comparative orotomy to dissect the components and tectonic histories of both the Archean Superior and Phanerozoic Altaid orogenic systems, finding remarkable similarities between them, including their rock types, sequence of structural progression, and spatial and temporal scales of evolution. From this, we conclude that the Archean Superior Province formed in much the same way that the crust of the Altaids formed, that is by the off-scraping of oceanic material, including ophirags, in subduction/accretionary complexes, through which arc magmatic fronts migrated, and which thickened during collisions. Both orogenic collages were sliced and rearranged along major strike-slip fault systems during and after their main phases of growth and deformation.

Comparative orotomy is thus a very powerful method to understand the origin of, especially, very ancient cratons or orogens, where tools such as fossils, paleomagnetism and plate reconstructions are typically lacking [9]. For instance, in another comparative study, T Kusky *et al*. [7] recently concluded that the Mesoarchean Pilbara Craton formed in much the same way, by early off-scraping of oceanic material, including ophirags, in a subduction/accretionary complex, that was later intruded by several generations of arc magmas with intervening deformation events that deformed the arc plutons into domes, then sliced by strike-slip faults. The extremely small size of the eastern Pilbara (200 × 200 km) was shown in comparison to the Sierra Nevada batholith, into which the entire eastern Pilbara dome-and-basin province fits, with similar rock types, structures, and spatial and temporal scales of development. Further, the central and western Pilbara, in that comparison, correlate (in terms of having a similar tectonic setting) with the Great Valley and Franciscan accretionary complexes of California, respectively.

By using comparative orotomy for the Superior Province, it becomes clear that narrow belts of plutonic domes with intervening basins of volcano-sedimentary deposits do not represent a unique vertical tectonic regime on Earth, where in the Archean some have postulated that dome-and-basins represent a stagnant lid drip or sagduction tectonic mode of early Earth dynamics (e.g. [130,184]), but these granitic and gneiss domes are located in fragments of subduction/accretionary complexes, intruded by arc plutons, where vertical intrusion is accompanied by horizontal strike-slip faulting [185]. Domal plutons, surrounded by sinking rafts of previously accreted material, and other syn-orogenic deposits are common in oceanic and continental-margin arc systems [8], so by comparison with modern arc systems, and comparing tectonic belts with similar affinities but different ages, it becomes clear that the processes that formed the domes of the Oxford Stull volcanic-plutonic domain of the Superior are no different from the domes of Phanerozoic arc systems.

Thus, we suggest that comparative orotomy is a powerful method to understand some of the world's oldest rocks, by searching for similar orogens or other settings to compare with, instead of formulating *ad hoc* histories for old rocks, which, in many cases, result in models that have no Earthly analogs, and are unlikely to be correct.

## Supplementary Material

nwac235_Supplemental_File
