# Supplementary material for: Comparative orotomy of the Archean Superior and Phanerozoic Altaid orogenic systems
Source: Natl Sci Rev. 2022 Oct 25;10(2):nwac235. doi: 10.1093/nsr/nwac235 (PMC9933457; doi:10.1093/nsr/nwac235)
Supplement: nwac235_Supplemental_File [file nwac235_supplemental_file.docx]

**Supplementary Information 1.**

**Comparative orotomy of the Archean Superior and Phanerozoic Altaid Orogenic systems**

T.M. Kusky^a,b,c^, A.M.C. Şengör^d,e,a^

^a^Center for Global Tectonics, School of Earth Sciences and State Key Laboratory for Geological Processes and Mineral Resources, China University of Geosciences, Wuhan 430074, China

^b^ Badong National Observatory and Research Station for Geohazards, Ministry of Education, China University of Geosciences, Wuhan 430074, China

^c^ Department of Geological Engineering, Middle East Technical University, Üniversiteler Mahallesi, Dumlupınar Bulvarı No. 1, Çankaya, TR, 06800 Ankara Türkiye,

^d^Eurasia Institute of Earth Sciences, Istanbul Technical University, Ayazağa,34469, Istanbul, Türkiye,

^e^Department of Geology, Faculty of Mines, Istanbul Technical University, Ayazağa, 34469, Istanbul, Türkiye,

**Supplementary Information 1.**

**Description of the geology and tectonic units of the Superior Province**

In this section we briefly review the geology and tectonics of the Superior Province, and refer the reader to a more comprehensive reviews by [1,2], which is the main source of data for the divisions of the craton used herein, albeit we have modified these classifications that were based on terranology, and redefined tectonic zones based on their principal geologic characteristics. All maps and locations referred to are keyed to Fig. 6.

**Western and Southern Superior Province**

The ancient *Hudson’s Bay domain* (Hudson’s Bay terrane of [2] in the NW part of the Craton (HBT on Fig. 6) consists of granites and various types of gneisses, and has detrital zircons of up to 3.9 Ga [3]. Different suites of intrusions are known from 3.2-3.1, 2.85-2.81, and 2.74-2.71 Ga [3]. The Hudson’s Bay terrane of [2] includes the Hudson’s Bay domain, includes one outcrop region at the northern exposed limit of the shield at its boundary with the Trans Hudson orogen in the northwest, west of Hudson’s Bay, and it continues across Hudson’s Bay as the *La Grande, Bienville, Goudalie, and Tikkerutuk domains* (Fig. 6). Since there is very little known about the details of the oldest rocks in the various domains east and west of Hudson’s Bay (with about 1000 km separating them), and the age differences are up to 1 Ga, we abandon the term Hudson’s Bay terrane and instead describe each domain separately. We define instead, the ancient continental-aspect Paleo-Mesoarchean belt on the NW side of Hudson’s Bay as the Hudson’s Bay domain, or refer to it as a ribbon continent when making interpretations, and speculate on any possible correlations later, outside of the definitions.

South of the Hudson’s Bay domain, the *Oxford-Stull and Island Lake domains* are volcano-plutonic subprovinces that represent subduction/accretionary complexes through which arc magmatic fronts have migrated. The Oxford-Stull domain consists of circa 2.88-2.73 juvenile continental margin volcanic-intrusive complex rocks imbricated with oceanic crustal fragments (ophirags) before 2.734 Ga [4], whereas the Island Lake Domain is dominantly plutonic with a few scattered volcano-sedimentary enclaves. These subduction/accretion/arc complexes are bordered to the south by the massive circa 2745-2708 Ma *Berens River TTG complex*, likely the root of a large arc system. The southern margin of the Berens River TTG complex is occupied by the *Uchi domain,* which has a long history spanning 300 Ma, with circa 3.0 Ga basement overlain by a 2990-2960 Ma rift sequence, followed by 2940-2910 Ma arc volcanics [5], in turn cut by 2870-2850 plutons, suggestive of a continental margin arc setting [2].

In the terrane classification of [2] the *North Caribou* *terrane* includes the *Oxford-Stull and Island Lake domains* in the north*, the Berens River domain in the core, and Uchi domain* (Fig. 6) on the south, along the western side of James Bay.

The > 800 km long linear dominantly metasedimentary graywacke/turbidite *English River Belt* marks the southern boundary of the Uchi/Berens River arcs, and was metamorphosed at 2.69-2.66 Ga. Detrital zircons have ages of 3.24-2.704 Ga, suggesting deposition after cessation of magmatic arc activity in the bounding North Caribou and Winnipeg River terranes, suggesting that the English River Belt is a metamorphosed syn-orogenic flysch basin, recording the amalgamation of the two arc terranes during the metamorphic peak at 2692 Ma, with a late thermal peak (post-orogenic extension?) at 2669 Ma [6,7].

To the south, gneissic and plutonic rocks of th**e** *Winnipeg River ribbon microcontinent* include 3.32-2.83 tonalites [8] overlain by a continental margin sequence [9]. Neoarchean magmatism and associated multi-phase deformation is documented from 2730-2690 Ma [10-11]. On its northern margin, the Bird River granite-greenstone belt is a small subduction/accretion/arc complex containing several ophirags, and associated with granitoid plutons with ages of 2858-2646 Ma [12] and exhibits greenschist to amphibolite facies metamorphism, folding, and shearing associated with convergence between the Winnipeg River and Uchi domain at 2720-2700 Ma. Several circa 2743 Ma intrusions including the Mayville, Euclid Lake, New Manitoba Mine and Cat Lake intrusions cut the volcano-sedimentary sequence [12], including the > 20 km long dismembered chromite-bearing Bird River sill [13]. Field relationships and geochemistry have been interpreted to show that the 2743Ma Bird River sill (Fig. 7A), consisting of pyroxenite, anorthosite, gabbro, layered chromitite, and diorite was emplaced into the greenstone belt in an oceanic setting [12] then intruded by the Maskawa Lake batholith. Major and trace element geochemisty show a suprasubduction origin, leading [12] to suggest that this is a dismembered subduction-related ophiolite formed at the transition from a continental arc to a back arc basin, and marking the suture with the Winnipeg River microcontinent. The Mayville intrusion (Fig. 7B) includes megacrystic layered anorthosites containing amphibole, indicating that it crystallized from a hydrated magma [12]. Trace element and Sm-Nd and U-Th-Pb isotopic systematics show that the pluton was derived by partial melting of a depleted shallow sub-arc mantle source, and that these plutons formed in an Andean-type continental margin arc with petrological similarities to modern Andean arcs [12].

The Winnipeg River ribbon continent forms an oroclinal structure similar to that of the Central Mongolian (Tuva-Mongol arc, unit 43.3 on Fig. 3) ribbon continent in the Altaids. The oroclinal core surrounds the W*estern Wabigoon subduction/accretion/arc complex* (Fig. 6), in a pattern remarkably similar to the Khangay-Khantey subduction/accretionary complex in the core of the Tuva-Mongolian arc’s oroclinal bend.

The W*estern Wabigoon subduction/accretion/arc complex* (WWD on Fig. 6) is dominated by circa 2745-2720 mafic volcanic rocks intruded by circa 2735-2720 Ma tonalites [14]. At Kagagi Lake (Figs. 6), circa 2723 Ga tholeiitic basalts and associated gabbroic to ultramafic complexes intruded by circa 2727 Ma trondhjemitic stocks form the oldest rocks in the basement, succeeded by a series of arc-like calc-alkaline andesites, dacites, and associated pyroclastic and sedimentary deposits, then folded and faulted together before intrusion of quartz-diorite plutons by circa 2480 Ma [14]. These ophirags are widely interpreted to represent a collage of oceanic crustal and plateau remnants, intruded by arc-related magmas [9,15-18]. This orogenic collage is locally overlain by narrow belts (strike slip basins?) of 2711-2702 Ma metasedimentary rocks, with detrital zircons as old as 3.0 Ga. These narrow basins are interpreted by [19,20] as small foredeeps formed in front of imbricated thrust nappes.

The *Marmion block* (MB on Fig. 6) has an older circa 3010-2999 Ma tonalitic basement and is likely a strike-slip displaced section of the Winnipeg River microcontinent [21], both of which have juvenile isotopic signatures of 3.0 Ga - 3.4 Ga [22]. The Marmion terrane is overlain by a spectacular <2710 Ma sedimentary sequence (Fig. S1) including basal clastics and a thick stromatolitic carbonate sequence [8,21,23,24], that was shown to be overthrust by stacks of volcanic sequences named the Witch Bay allochthon, and interpreted as a juvenile arc sequence by [21] along its southern margin [25,26]. These, and their correlatives, may be related to the oceanic/arc assemblages of the Western Wabigoon subduction/accretionary/arc assemblage.


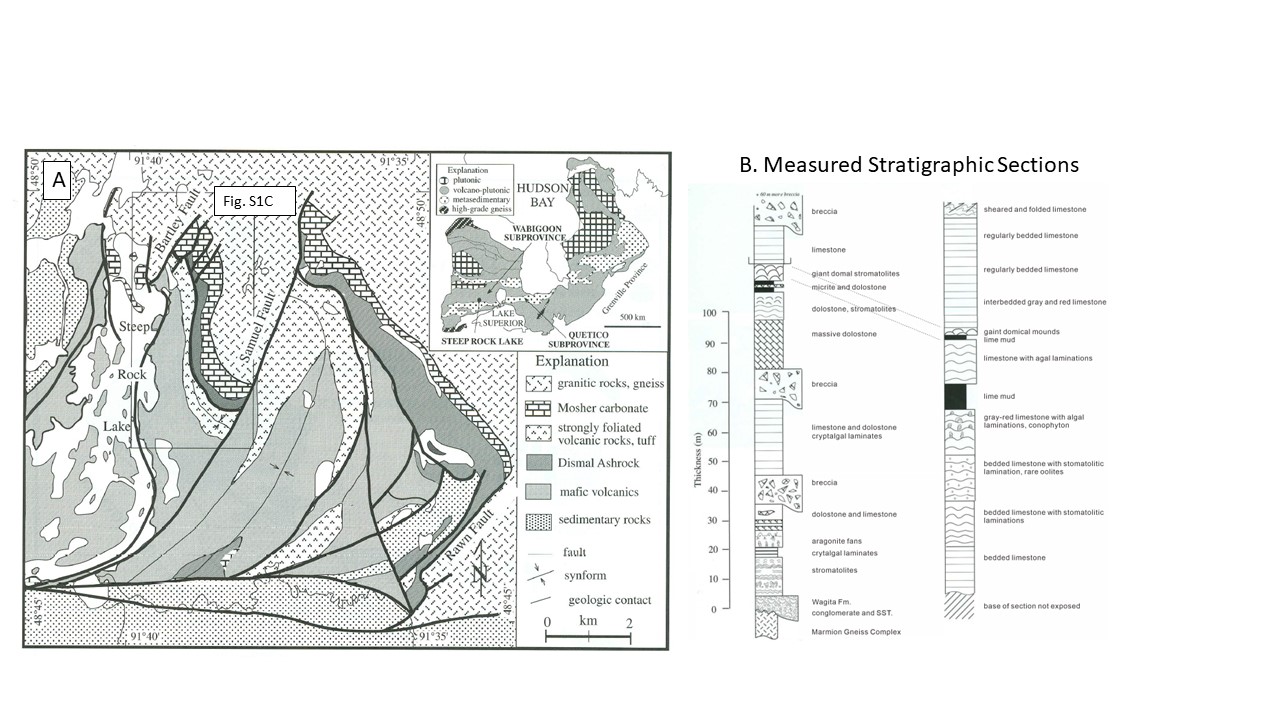


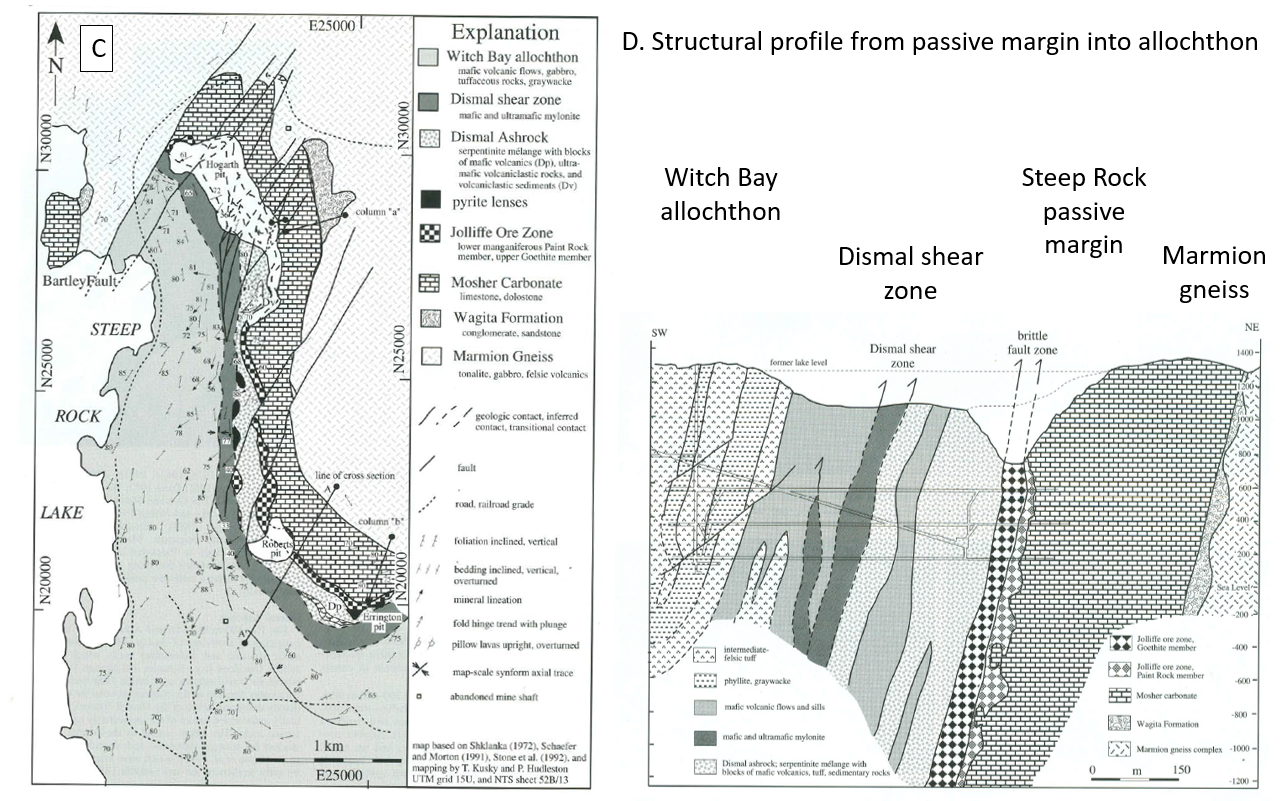


***Figure S1.*** *Geologic map (A) and measured stratigraphic profiles through the Steep Rock passive margin (modified from* [21]*). Locations of stratigraphic sections are shown on the detailed structural map (C) of the Hogarth pit (mapped when it was drained), and (D) cross section from the basement Marmion gneiss (3.01 Ga) through the passive margin, into the Dismal shear zone and the overlying Witch Bay volcanics, consisting of pillow lavas and arc-volcanics.*

The *Quetico* (**Que**bec **Ti**mber **Co**mpany) belt (QB on Fig. 6) forms the longest straight and relatively narrow Neoarchean metasedimentary belt in the Superior craton (and hence the world). It consists of poly-deformed graywacke, migmatites (derived from graywacke) intruded by granitoids [27,28]. Sedimentation in the belt is constrained to be between 2698-2696 Ma [29], or <2692 Ma [30], during the collision of the amalgamated subduction/accretion/arc complexes, and the Winnipeg River/Marmion ribbon continent to the north with the large Wawa-Abitibi oceanic plateau to the south[2]. This was followed by the intrusion of Alaskan-style mafic-ultramafic complexes [31] followed by low-P high-T amphibolite-granulite facies metamorphism and accompanying granitic magmatism at 2670-2650 Ma [32]. The northern margin of the Quetico belt is marked by the dextral, circa > 1000 km long Quetico strike-slip fault [33], that likely records major strike-slip structural slicing and repetition of different arc complexes of the Superior Province by 2650 Ma, in the late stages of formation of the craton [21]. The Quetico fault system is one of the largest (>1000 km) Archean transcurrent fault systems on Earth, which [34] noted as one line of evidence to support lateral plate motions in the Archean.

The oceanic-derived *Wawa-Abitibi subprovince* forms a huge complex of accreted oceanic and arc type magmatic rocks south of the Quetico and Opatica belts [35], dominating the map pattern of the southern Superior Province (WAC on Fig. 6). In some places boundaries between different smaller tectonic units including ophirags are marked by tectonic mélanges [36]. Late volcanism at circa 2695 is associated with early thrusting and incorporation of the different ophiolitic and arc assemblages into an accretionary wedge type setting (e.g., 18,36,37), followed by later-stage calc-alkaline to alkaline magmatism, transpressive deformation, and deposition of coarse clastic sediments during the so-called Shebandowanian phase of the Kenoran orogeny [38] at 2685-2680 Ma. The Wawa-Abitibi terrane is well-studied in its central and eastern sectors, partly driven by an abundance of gold deposits associated with specific structures and volcano-sedimentary sequences, such as the Timiskaming [38]. Eastern parts of the Wawa-Abitibi terrane show oceanic-affinity basement with no isotopic inheritance [39]. There are a variety of ideas for the evolution of the Wawa-Abitibi terrane, but all must explain the changing nature of volcanism from 2790-2680 Ma [2,40] (Percival et al., 2012; personal communication, 2022). Many models suggest that the volcanism suggests evolution from early oceanic MORB or plateau type volcanism, to arc then eventually rifted arc environments [41,42]. The volcanic sequences are overlain by a regional sequence of circa < 2690 Ma graywacke sequence [43] (Porcupine Group [39]), then the < 2672 Timiskaming Group [44], formed in basins associated with late strike-slip faults such as the major Quetico fault. The southern boundary of the Wawa-Abitibi belt is the Cadillac- Larder Lake “break”, a south-vergent thrust that emplaced the Abitibi belt over the Pontiac belt to the south.

Because of glacial cover in the southern Superior craton, the *Pontiac belt* (PB on Fig. 6) is not as well-known as areas further north, but it consists of metasedimentary schists and paragneiss with sources up to 3.0 Ga, and depositional ages of < 2685 Ma [45]. Structural studies (e.g., [44]) suggest that the Pontiac belt is a south-vergent fold-thrust belt.

The Minnesota River Valley gneiss complex (MRV on Fig. 6), where exposed, contains some of the oldest rocks in the Superior Province, with ages as old as 3.5 Ga [46]. The ancient gneiss complex includes migmatitic granitic gneisses, schistose to gneissic amphibolite and metagabbro, and paragneisses including the circa 3485 Ma Montevideo gneiss, and the 3524 Ma Morton gneiss, strongly metamorphosed at circa 2600 Ma [46]. Seismic imagery shows it was thrust beneath the Wawa-Abitibi terrane, with a collision at 2685 Ma, after which it was covered by turbidites from 2685-2682 Ma, and intruded by granites at 2670-2650 Ma [2]. We interpret the Minnesota River Valley gneisses as the tip of a southern ribbon continent, following [46].

**Eastern Superior Province**

In the eastern Superior Province, the *Opatica belt* borders the north of the Wawa-Abitibi subduction/accretion complex (O on Fig. 6). This is another subduction/accretion/arc complex that consists of circa 2820 Ma tonalite, 2770-2700 Ma tonalite-granodiorite and 2680 Ma granites [47], with less-common 2790-2750 Ma greenstone belts such as the Frotet-Evans belt [48] that, because of the presence of boninites, has been interpreted to represent an extended forearc ophiolite [49]. The boninitic rocks occur within a thrust sheet of transitional tholeiitic basalts, magnesian- and ferro-tholeiites (Fig. S2), interpreted by [49] to represent a marginal basin or forearc ophiolite formed by fore-arc extension related to slab rollback above a N-dipping subduction zone, remnants of which are still visible in the structure revealed by seismic reflection [50]. Polyphase deformation at >2690 and 2690-2680 Ma has dismembered the ophiolite into separate ophirags (Fig. S2), formed a series of basins and domes, and west- and south-vergent structures [50,51].

The *Opinaca belt* (OP on Fig. 6) includes low-grade polydeformed graywacke, conglomerate, iron formation and mafic rocks on the edges of the belt, whereas interior portions of the belt are metamorphosed to amphibolite-granulite facies.


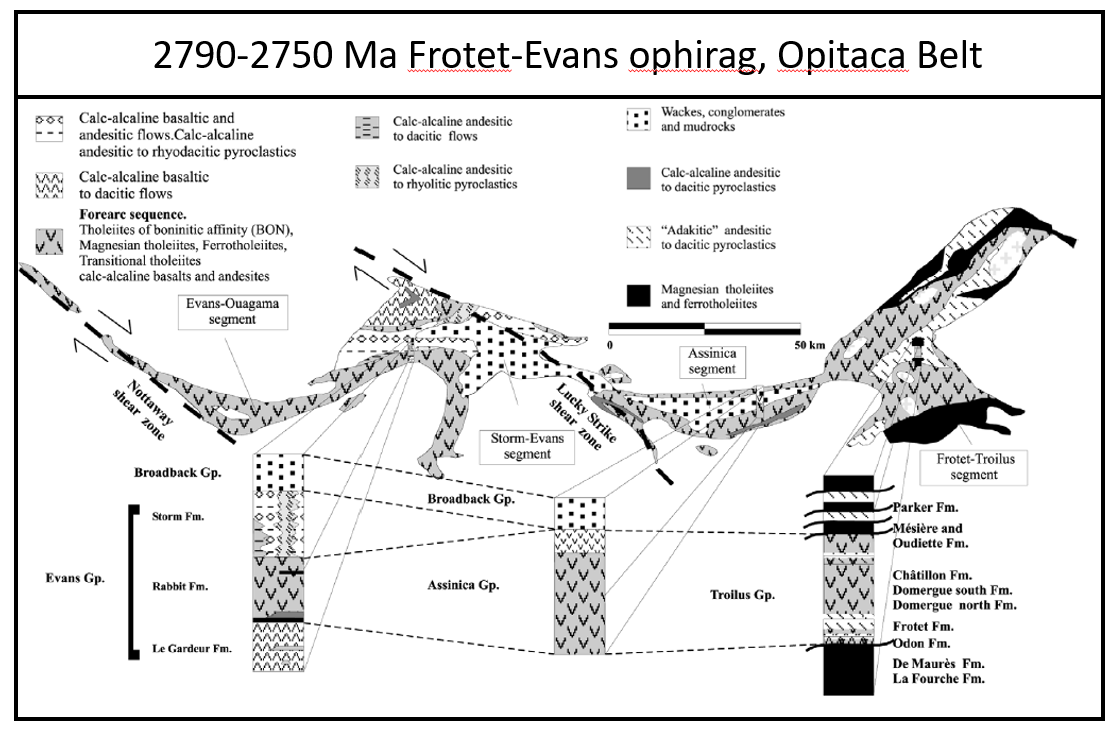


***Figure S2.*** *Map of* *the Frotet-Evans greenstone belt, Opitaca Belt (see Fig. 6 for location). Rocks of the lower De Maures, LaFource, Rabbit, and other formations form lower thrust sheets that include transitional tholeiites, magnesian tholeiites, ferrotholeiites, and boninitic rocks (shown in black), interpreted as an extended forearc ophiolitic sequence (map modified from* [49]*.*

The southern part of the *La Grande belt* to the north (LG on Fig. 6) was thrust over the Opinaca belt forming conglomerate-filled foredeeps, with detrital zircons showing thrusting occurred after 2.65 Ga [52]. This belt contains a sequence of juvenile 2720-2700 Ma volcanic dominated arc-type rocks in the *Eastmain belt* (E on Fig. 6) including komatiitic, tholeiitic, calc-alkaline and adakitic rocks [53,54]. The northern part of the La Grande Belt is different in character, appearing older and more evolved, but data is not sufficient to determine it tectonic affinity or whether it is an “essential” or “accidental” tectonic unit.

**Northeastern Superior Province**

The tectonic belts north and east of Hudson’s Bay strike north-northwest, strikingly different from the general east-west strike of the southern and western Superior Province tectonic belts, forming an oroclinal structure similar in scale and perhaps origin and significance to the great oroclinal bend of the Altaids (Fig. 1). In older terrane-based tectonic models, some of the tectonic belts of the NE Superior Province were correlated with the Hudson’s Bay domain in the west [2], but the huge discrepancies in ages, rock types, and degree of knowledge, leads us to abandon this correlation and grouping into terranes, and just describe the tectonic units based on what they contain, and their histories.

In the northern and eastern *La Grande domain* (Fig. 6), Mesoarchean basement is locally overlain by quartz arenites and 2750-2730 Ma volcanic rocks, and circ 2820 Ma komatiites [55,56]. The *Bienville domain* includes TTG gneisses and 2.74-2.69 Ga granodioritic-granitic intrusives, whereas the *Tikkerutuk domain* consist of circa 2730-2690 Ma tonalite, diorite, and enderbite intruded by 2720-2690 Ma granites. Enclaves of older tonalitic gneisses have ages of up to 3.02 Ga [57]. Isolated volcano-sedimentary belts in the northern part of the terrane mostly have ages of 2.76-2.70 Ga, although the Nuvvuagittuq belt has yielded ages of 3.8-3.6 Ga [58] and possibly up to 4.3 Ga [59-63].

**Tikkerutuk Domain- Earth’s oldest vestige of oceanic arc accretion**

The *Tikkerutuk domain* (T on Fig. 6) east of Hudson’s Bay consists largely of Neo-Archean plutonic rocks, but also has rare Eo-Mesoarchean rocks including charnockites with narrow intervening volcano-sedimentary sequences [58]. Out of the dozen or so known volcano-sedimentary enclaves within TTG gneisses of the Tikkerutuk domain, the best known is the small (10 km^2^) > 3.8 Ga Nuvvuagittuq belt (Fig. 8), that includes oceanic-affinity ultramafic, gabbroic, and amphibolitic units including basaltic pillows, bedded cherts, and iron formations, encapsulated as one of several enclaves in a sea of circa 3.66-3.8 Ga tonalite-trondhjemite-granodiorite-granite-monzonite intrusions [60-62,64]. The pillowed mafic volcanics and associated rocks have ages of 3750-4280 Ma [63], interpreted to represent a complexly refolded shred of early oceanic crust (the world’s oldest ophirag) and overlying hydrothermal precipitates of an ocean-plate stratigraphic section [66-69]. Interestingly, these oldest-known sea-floor hydrothermal deposits contain “putative fossils” [65], showing that the oldest oceanic crust was host to anoxygenic phototrophs that contributed to the formation of sea floor BIF’s by ferrous iron oxidation before the Great Oxidation Event [65].

The *Goudalie domain* (G on Fig. 6) includes several small volcano-sedimentary belts such as the Vizien greenstone belt [70], which contains structurally interleaved 2725 Ma continental arc rocks and 2790 ophirags [71], consisting of pillowed low-K tholeiitic andesites, komatiites, peridotites, and gabbro sills (Fig. 9). This leads us to classify the Goudalie domain as a vestige of a former ocean (a subduction/accretion/arc complex), instead of a “high-grade” terrane as in the classification of [2]. Within the Goudalie domain, the Vizien ophirag was interpreted as a highly-deformed sliver of an allochthonous oceanic plateau [72] based on its LREE-depletion indicating extraction from a depleted mantle source. [71] showed, through detailed structural analysis, that the ophirag (Fig. 9) was emplaced, along with the formation of a thin ophiolitic mélange along the thrust during D1, then deformed into an accretionary-wedge style refolded nappe (Fig. 9), and folded and faulted up to five times before attaining its present geometry. When unfolded, its geometry is revealed to be a typical accretionary wedge structure, involving ~2.8 Ga ophiolitic arc-type basement thrust over an older (circa 3.1 Ga) arc-like basement sequence (Fig. 9 B,C).

The high-grade metamorphic/plutonic *Ashuanipi complex* (A on Fig. 6) forms the easternmost part of the Superior craton. It is roughly 300 x 300 km in dimensions (Fig. 6) and consists of paragneisses with detrital zircons of 3.0-2.7 Ga, iron formation, circa 2725 Ma tonalite and 2.71 Ga volcanics, all metamorphosed up to granulite facies at 2680-2570 Ma [2,73]. It may represent high-grade metamorphic equivalents of the surrounding Opinaca and Opatica terranes [74].

In the extreme north, the 2.9-2.8 Ga Riviere Arnaud terrane of [2] based on [75-77] was proposed to include the Lac Minto, Qalluviartuuq, Utsalik and Douglas Harbour domains. Since this “terrane” model yields boundaries that cross geological/structural contacts, includes suites of rocks so diverse that they likely have little to do with each other, and the database is small, we do not accept this classification, and abandon it, focusing instead on the geological characters of the better-defined tectonic belts in the NE Superior Province [2].

The *Lac Minto domain* (subduction/accretion/arc complex; LM on Fig. 6) includes paragneiss and migmatite with hb- and pyx bearing 2730-2690 Ma granite intrusions [78]. Greenstones (highly deformed and metamorphosed ophirags) include the Kogaluc Belt [70] with <2748 Ma mafic volcanics and graywacke. The *Qalluviartuuq domain* (Q on Fig. 6) contains several oceanic greenstone belts (including the Payne Lake, Qalluviartuuq and Duquet belts), encapsulated in coeval 2850-2770 Ma tonalitic-trondhjemitic plutons, intruded by TTG at 2760-2710 Ma, and granite at 2730-2670 Ma [79], so is also an accretionary/ subduction complex intruded by trench-migrating arc magmatism. Although these domains are metamorphosed to generally high-grade and were classified as “metamorphic domains or terranes” in the above classifications, we adhere to classifying them according to their original character, which is that of accretionary/subduction/arc complexes (Fig. 6).

The *Utsalik domain* (U on Fig. 6) consists of sheets of circa 2740-2690 Ma enderbite, granodiorite and granite and rare volcano-sedimentary belts and tonalitic gneisses [80]. On the NE coast, the *Douglas Harbour domain* (DH on Fig. 6) contains volcano-plutonic complexes [81], suggesting that this region is transitional into a continental arc terrane.

**Supplementary References**

1. Percival, J.A., Sanborn-Barrie, M., Stott, G. et al. Tectonic evolution of the Western Superior Province from NATMAP and LITHOPROBE studies: *Canadian Journal of Earth Sciences* 2006; **43**: 1085–1117.

2. Percival, J.A., Skulski, T., Sanborn-Barrie, M. et al. Geology and Tectonic Evolution of the Superior Province, Canada. In: Percival et al. (Eds.), Tectonic Styles in Canada: The LITHOPROBE Perspective. *Geological Association of Canada, Special Paper* 2012; **49**: 321–378.

3. Skulski, T., Percival, J.A. Whalen, J.B., and Stern, R.A. Archean crustal evolution in the northern Superior Province. In Tectonic and Magmatic Processes in Crustal Growth: A Pan-LITHOPROBE Perspective. LITHOPROBE Secretariat, University of British Columbia, *LITHOPROBE Report* 1999; **75**: 128–129.

4. Stone, D., Corkery, M.T., Halle, J., Ketchum, J., Lange, M., Skulski, T., and Whalen, J. Geology and tectonostratigraphic assemblages, eastern Sachigo Subprovince, Ontario and Manitoba. *Ontario Geological Survey,* Preliminary Map P.3462 (also *Manitoba Geological Survey Open File OF 2003-2 and Geological Survey of Canada*, scale: 1:250 000: 2004; Open File 1582,

5. Sanborn-Barrie, M., Skulski, T., and Parker, J.R. Three hundred million years of tectonic history recorded by the Red Lake greenstone belt, Ontario. *Geological Survey of Canada; Paper* 2001: **2001-C19**.

6. Corfu, F., Stott, G.M., and Breaks, F.W. U-Pb geochronology and evolution of the English River subprovince, an Archean low P - high T metasedimentary belt in the Superior Province. *Tectonics* 1995; **14:** 1220–1233.

7. Haynes, J.A., and Archibald, D.A. Post-orogenic tectonothermal history of the Archean western Superior Province of the Canadian Shield as determined by conventional and laser Ar-Ar data: new data. InWestern Superior Transect Sixth Annual Workshop. Edited by R.M. Harrap and H. Helmstaedt. LITHOPROBE Secretariat, University of British Columbia, *LITHOPROBE* **Report 80**; 2001: 26–28.

8. Tomlinson, K.Y., Stott, G.M., Percival, J.A. et al. Basement terrane correlations and crustal recycling in the western Superior Province: Nd isotopic character of granitoid and felsic volcanic rocks in the Wabigoon sub- province, N. Ontario, Canada. *Precambrian Research* 2004; **132**: 245–274.

9. Sanborn-Barrie, M. and Skulski, T. Sedimentary and structural evidence for 2.7 Ga continental arc-oceanic arc collision in the Savant - Sturgeon greenstone belt, western Superior Province, Canada. *Canadian Journal of Earth Sciences* 2006; **43**: 995–1030.

10.Tomlinson, K.Y., Davis, D.W., Percival, J.A. et al. Mafic to felsic magmatism and crustal recycling in the Obonga Lake greenstone belt, Western Superior Province: evidence from geochemistry, Nd isotopes and U-Pb geochronology. *Precambrian Research* 2002; **114:** 295–325.

11. Melnyk M.J., Cruden, A.R., Davis, D.W. et al. U-Pb ages constraining regional deformation in the Winnipeg River subprovince and Lake of the Woods greenstone belt: Evidence for Archean terrane accretion in the western Superior Province. *Canadian Journal of Earth Sciences* 2006; **43**: 967–993.

12. Sotiriou, P., Polat, A., Frei, R. et al. Evidence for Neoarchean hydrous arc magmatism, the anorthosite‐bearing Mayville Intrusion, western Superior Province, Canada. *Lithos* 2020; **362‐363**: 105482.

13. Yang, X.M. and Gilbert, H.P. Mineral chemistry of chromite in the Mayville intrusion: evidence for petrogenesis and linkage to the Bird River sill in the Neoarchean Bird River greenstone belt, southeastern Manitoba (NTS 52L5, 6, 12); in *Report of Activities* 2014, Manitoba Mineral Resources, Manitoba Geological Survey, 2014: 32–48.

14. Davis, D.W. and Edwards, G.R. Crustal evolution of Archean rocks in the Kakagi Lake area, Wabigoon Subprovince, Ontario, as interpreted from high precision U-Pb geochronology. *Canadian Journal of Earth Sciences*, 1986; **23**: 182–192.

15. Davis, D.W., Krogh, T.E., Hinzer, J., and Nakamura, E. Zircon dating of polycyclic volcanism at Sturgeon Lake and implications for base metal mineralization. *Economic Geology* 1986; **80**: 1942–1952.

16. Blackburn, C.E., John, G.W., and Ayer, J. et al. Wabigoon Subprovince. In Geology of Ontario. Edited by P.C. Thurston, H.R. Williams, R.H. Sutcliffe, and G.M. Stott 1991; *Ontario Geological Survey,* Special Volume 4, Part 1; 303 –381.

17. Kusky, T.M., Polat, A., Growth of granite-greenstone terranes at convergent margins, and stabilisation of Archean cratons. *Tectonophysics* 1999; **305**: 43-73.

18. Wyman, D., Ayer, J., and Devaney, J. Niobium-enriched basalts from the Wabigoon subprovince, Canada: evidence for adakitic metasomatism above an Archean subduction zone. *Earth and Planetary Science Letters* 2000; **179**: 21–30.

19. Davis, D.W., Sutcliffe, R.H., and Trowell, N.F. Geochronological constraints on the tectonic evolution of a late Archean greenstone belt, Wabigoon subprovince, northwest Ontario. *Precambrian Research* 1988; **39**: 171–191,

20. Poulsen, K.H., Borradaile, G.J., and Kehlenbeck, M.M. An inverted Archean succession at Rainy Lake, Ontario. *Canadian Journal of Earth Sciences* 1980; **17**: 1358–1369.

21. Kusky, T.M., and Hudleston, P.J. Growth and Demise of an Archean carbonate platform, Steep Rock Lake, Ontario Canada. *Canadian Journal of Earth Sciences* 1999; **36**:1-20.

22. Tomlinson, K.Y., Davis, D.W., Stone, D. et al. U–Pb age and Nd isotopic evidence for Archean terrane development and crustal recycling in the south-central Wabigoon subprovince, Canada. *Contributions to Mineralogy and Petrology* 2003; **144**: 684–702.

23. Joliffe, A.W. Geology and iron ores of Steep Rock Lake, *Economic Geology* 1955; **50**: 373-398.

24. Fralick, P., and Riding, R. Steep Rock Lake: Sedimentology and geochemistry of an Archean carbonate platform, *Earth Science Reviews* 2015; **151**: 132-175,

25. Devaney, J.R. and Williams, H.R. Evolution of an Archean subprovince boundary: a sedimentological and structural study of part of the Wabigoon-Quetico boundary in northern Ontario. *Canadian Journal of Earth Sciences* 1989; **26**: 1013–1026.

26. Tomlinson, K.Y., Hall, R.P., Hughes, D.J. et al. Geochemistry an assemblage accretion of metavolcanics rocks in the Beardmore-Geraldton greenstone belt, Superior Province. *Canadian Journal of Earth Sciences* 1996; **33**: 1520–1533.

27. Percival, J.A. A regional perspective of the Quetico metasedimentary belt, Superior Province, Canada. Canadian Journal of Earth Sciences, 1989; **26**: 677–693.

28. Williams, H.R. Quetico subprovince. In Geology of Ontario. Edited by P.C. Thurston, H.R. Williams, R.H. Sutcliffe, and G.M. Stott. *Ontario Geological Survey* 1991; Special Volume 4, Part 1: 383–403.

29. Davis, D.W., Pezzuto, F., and Ojakangas, R.W. The age and provenance of metasedimentary rocks in the Quetico subprovince, Ontario, from single zircon analyses: Implications for Archean sedimentation and tectonics in the Superior Province. *Earth and Planetary Science Letters* 1990; **99**: 195–205.

30. Zaleski, E., van Breemen, O., and Peterson, V.L. Geological evolution of the Manitouwadge greenstone belt and Wawa-Quetico subprovince boundary, Superior Province, Ontario, constrained by U-Pb zircon dates of supracrustal and plutonic rocks. *Canadian Journal of Earth Sciences* 1999; **36:** 945–966.

31. Pan, Y., Fleet, M.E., and Heaman, L.M. Thermo-tectonic evolution of an Archean accretionary complex: U-Pb geochronological constraints on granulites from the Quetico Subprovince, Ontario, Canada. *Precambrian Research* 1998; **92**: 117–128.

32. Pettigrew, N.T. and Hattori, K.H. The Quetico Intrusions of western Superior Province: Neo-Archean examples of Alaskan/Ural-type mafic–ultramafic intrusions: *Precambrian Research* 2006; **149**: 21–42.

33. Tabor, J.R., and Hudleston, P. Deformation at an Archean sub-province boundary, northern Minnesota, *Canadian Journal of Earth Sciences* 1991; **28**: 292-307.

34. Kusky, T.M., Windley, B.F., Polat, A. Geological evidence for the operation of plate tectonics throughout the Archean: Records from Archean plate-boundaries. *Journal of Earth Sciences* 2018; **29**: 1291-1303.

35. Williams, H.R. Quetico subprovince. In Geology of Ontario. Edited by P.C. Thurston, H.R. Williams, R.H. Sutcliffe, and G.M. Stott. *Ontario Geological Survey* 1991; Special Volume 4, Part 1: 383–403.

36. Polat, A. and Kerrich, R. Formation of an Archean tectonic melange in the Schreiber-Hemlo greenstone belt, Superior Province, Canada; implications for Archean subduction- accretion process. Tectonics 1999; **18**: 733–755.

37. Polat, A. The geochemistry of Neoarchean (ca. 2700 Ma) tholeiitic basalts, transitional to alkaline basalts, and gabbros, Wawa Subprovince, Canada: Implications for petrogenetic and geodynamic processes. Precambrian Research 2009; **168**: 83–105.

38. Davis, D.W. and Lin, S. Unraveling the geologic history of the Archean Hemlo gold deposit, Superior Province, Canada: A U-Pb geochronological study. Economic Geology 2003; **98**: 51–67.

39. Ayer, J.A., Thurston, P.C. and Bateman, R. et al. Overview of results from the greenstone architecture project: Discover Abitibi Initiative. *Ontario Geological Survey*, Open File Report 2005; 6154.

40. Stott, G.M. and Corfu, F. Uchi subprovince. In Geology of Ontario. Edited by P.C. Thurston, H.R. Williams, R.H. Sutcliffe, and G.M. Stott. *Ontario Geological Survey* 1991; Special Volume 4, Part 1: 145–238.

41. Thurston, P.C. Archean volcanic patterns. In Archean Crustal Evolution. Edited by K.C. Condie. Elsevier, Amsterdam, Developments in *Precambrian Geology* 1994; **11**: 45–84.

42. Wyman, D. and Kerrich, R. Mantle plume volcanic arc interaction: consequences for magmatism, metallogeny, and cratonization in the Abitibi and Wawa subprovinces, Canada. Canadian Journal of Earth Sciences 2010; **47**: 565–589.

43. Ayer, J.A., Thurston, P.C. and Bateman, R. et al. Overview of results from the greenstone architecture project: Discover Abitibi Initiative. *Ontario Geological Survey*, Open File Report 2005; 6154.

44. Davis, D.W. U-Pb geochronology of Archean metasedimentary rocks in the Pontiac and Abitibi subprovinces, Quebec, constraints on timing, provenance and regional tectonics. *Precambrian Research* 2002; **115**: 97–117.

45. Mortensen, J.K., and Card, K.D. U-Pb age constraints for the magmatic and tectonic evolution of the Pontiac subprovince, Quebec. *Canadian Journal of Earth Sciences* 1993; **30**: 1970-1980.

46. Bickford, M.E., Wooden, J.L., and Bauer, R.L. SHRIMP study of zircons from the Early Archean rocks in the Minnesota River Valley: Implications for the tectonic history of the Superior Province. Geological Society of America Bulletin 2006; **118**: 94–108.

47. David, J. and Parent, M. Geochronologie U-Pb du Projet Moyen-Nord. Ministere des Ressources naturelles, Quebec 1997; GM59903.

48. Rheaume, P., Bandyayera, D., Fallara, F., Boudrias, G., and Cheng, L.Z. Geologie et metallogenie du secteur du lac aux Loutres, synthese metallogenique d’Urban-Barry (phase 1 et 2). Geologie Quebec 2004; RP 2004-05.

49. Boily, M. and Dion, C. Geochemistry of boninite-type volcanic rocks in the Frotet-Evans greenstone belt, Opatica Subprovince, Quebec; implications for the evolution of Archaean greenstone belts. Precambrian Research 2002; **115**: 349–371.

50. Calvert, A., Sawyer, E.W., Davis, W.J., and Ludden, J.N. Archaean subduction inferred from seismic images of a mantle suture in the Superior Province. Nature 1995; **375**: 670–674.

51. Sawyer, E.W. Formation and evolution of granite magmas during crustal reworking: the significance of diatexites. Journal of Petrology 1998; **39**: 1147–1167.

52. Goutier, J., Dion, C., Ouellet, M.-C., Davis, D.W., David, J., and Parent, M. Geologie de la region du lac Guyer (33G/05, 33G/06, 33G/11). *Ministere des Resources naturelles*, Quebec 2002; RG 2001-15.

53. Skulski, T., Hynes, A., and Francis, D. Basic lavas of the Archean La Grande greenstone belt: products of polybaric fractionation and crustal contamination. Contributions to Mineralogy and Petrology 1988; **100**: 236–245.

54. Moukhsil, A., Legault, M., Boily, M., Doyon, J., Sawyer, E., and Davis, D.W. Synthese geologique et metallogenique de la ceinture de roches vertes de la Moyenne et de la Basse-Eastmain (Baie-James). *Ministere des Ressources naturelles,* Quebec 2003: ET 2002-06.

55. Roscoe, S.M. and Donaldson, J.A. Uraniferous pyritic quartz pebble conglomerate and layered ultramafic intrusions in a sequence of quartzite, carbonate, iron formation and basalt of probable Archean age at Lac Sakami, Quebec. In *Current Research, Part C*. Geological Survey of Canada 1988; **Paper 88-1C**: 117– 121.

56. St-Seymour, K. and Francis, D. Magmatic interaction between mantle and crust during the evolution of the Archean Lac Guyer greenstone belt. *Canadian Journal of Earth Sciences* 1988; **25**: 691–700.

57. David, J., Parent, M., Stevenson, R., Nadeau, P., and Godin, L. The Porpoise Cove supracrustal sequence, Inukjuak area: A unique example of Paleoarchean crust (ca. 3.8 Ga) in the Superior Province. *Ministere des Ressources Naturelles du Quebec* 2022; DV 2002–10.

58. Cates, N.L. and Mojzsis, S.J. Metamorphic zircon, trace elements and Neoarchean metamorphism in the 3.75 Ga Nuvvuagittuq supracrustal belt, Quebec (Canada). *Chemical Geology* 2009; **261**: 98–113.

59. O'Neil, J., Carlson, R.W., Francis, D., and Stevenson, R.K. Neodynium-142 evidence for Hadean mafic crust. *Science* 2008; **321**: 1828–1831.

60. O’Neil, J., Francis, D., Carlson, R.W. Implications of the Nuvvuagittuq greenstone belt for the formation of Earth’s early crust. *Journal of Petrology* 2011; **52**, 985–1009.

61. O’Neil, J., Carlson, R.W., Paquette. J-L., Francis, D. Formation age and metamorphic history of the Nuvvuagittuq greenstone belt. *Precambrian Research* 2012; **220-221**: 23-44.

62. Mloszewaka, A.M., Mojzsis, S.J., Pecoits, E., Papineau, D., Dauphas, N., Konhauser, K.O. Chemical sedimentary protoliths in the <3.75 Ga Nuvvuagittuq supracrustal belt (Québec, Canada). *Gondwana Research* 2013; **23**: 574 594.

63. Papineau, D., She, Z.B., Dodd, M.S. et al. Metabolically diverse primordial microbial communities in Earth’s oldest seafloor-hydrothermal jasper, *Science Advances* 2022; 8**:** 15.

64. Guitreau, M., Blichert-Toft, J., Mojzsis, S.J., Roth, A.S.G., Bourdon, B. A legacy of Hadean silicate differentiation from Hf isotopes in Eoarchean rocks of the Nuvvuagittuq supracrustal belt (Qu´ebec, Canada). *Earth and Planetary Science Letters* 2013; **362**: 171–181.

65. Dodd, M.S., Papineau, D., Grenne, T., Slack, J.F., Rittner, M., Pirajno, F., O’Neill, J., Little, C.T.S. Evidence for early life in Earth’s oldest hydrothermal vent precipitates. *Nature* 2017; **543**: 60–64.

66. Windley, B.F., Kusky, T.M., and Polat, A., Onset of plate tectonics by the Eoarchean. *Precambrian Research* 2021; **352**: 105980.

67. Kusky, T.M., Windley, B.F., Safonova, I. et al. Recognition of Ocean plate stratigraphy in accretionary orogens through Earth history: A record of 3.8 billion years of sea floor spreading, subduction, and accretion, GR Focus review paper, in Kusky, T.M., Stern, R.J., and Dewey, J.F., Secular Changes in Geologic and Tectonic Processes, Special Issue of *Gondwana Research* 2013; **42**: 501-547.

68. Kusky, T.M., Windley, B.F., Polat, A., et al. Archean dome-and-basin style structures form during growth of intraoceanic and continental margin arcs and their death by slab failure and collision, *Earth-Science Reviews* 2021; **220**:103725.

69. Papineau, D., She, Z.B., Dodd, M.S. *et al*. Metabolically diverse primordial microbial communities in Earth’s oldest seafloor-hydrothermal jasper, *Science Advances* 2022; **8**: 15.

70. Skulski, T. and Percival, J.A. Allochthonous 2.78 Ga oceanic plateau slivers in a 2.72 Ga continental arc sequence; Vizien greenstone belt, northeastern Superior Province, Canada. *Lithos* 1996; **37**: 163–179.

71. Lin, S., Percival, J.A., Winsky, P.A., Skulski, T., and Card, K.D. Structural evolution of the Vizien and Kogaluc greenstone belts in Minto block, northeastern Superior Province, northern Quebec, in *Current Research* 1995-C; Geological Survey of Canada 1995; 121-130.

72. Skulski, T. and Percival, J.A. Allochthonous 2.78 Ga oceanic plateau slivers in a 2.72 Ga continental arc sequence; Vizien greenstone belt, northeastern Superior Province, Canada. *Lithos* 1996; **37**: 163–179.

73. Percival, J.A., Stern, R.A., and Rayner, N. Archean adakites from the Ashuanipi Complex, eastern Superior Province, Canada: geochemistry, geochronology and tectonic significance. *Contributions to Mineralogy and Petrology* 2003; **145**: 265–280.

74. Leclair, A., Lamothe, D, Choiniere, J., and Parent, M. Geologie de la region du Lac Bermen (SNRC 23F). *Ministere des Ressources naturelles*, Quebec 1998; RG 97-11.

75. Leclair, A. Geologie du nord-est de la Province de Superieur. *Ministere des Ressources naturelles*, Quebec 2005; DV 2004-04, 21 p., echelle 1:750,000.

76. Boily, M., Leclair, A., Maurice, C. and Berclaz, A. et al. Etude geochimique et isotopique du Nd des assemblages volcaniques et plutoniques du nord-est de la Province du Supieur (NEPS). Ministere des Ressources naturelles, Quebec 2006; GM62031.

77. Stott, G.M., Corkery, M.T., Percival, J.A., Simard, M., and Goutier, J. A revised terrane subdivision of the Superior Province. In Summary of Field Work and Other Activities 2010. *Ontario Geological Survey*, 2010; Open File Report 6260, pp. 20-1 – 20-10.

78. Simard, M., Cheve, S., David, J. et al. Geologie de la region du lac Minto (34F et 34G). *Ministere des Ressources naturelles*, Quebec 2005; RG 2004-04.

79. Berclaz, A., Maurice, C., Lacoste, P., David, J., Leclerc, F., Sharma, K.N.M., Labbe, J.-Y., Goulet, N., Bedard, J., and Vallieres, J. 2005. Geology of the Lac Anuc Area (34O). *Ministere des Ressources naturelles*, Quebec 2005; RG 2003-05.

80. Percival, J.A., Stern, R.A., and Skulski, T. Crustal growth through successive arc magmatism: Reconnaissance U-Pb SHRIMP data from the northeastern Superior Province, Canada. *Precambrian Research* 2001; **109**: 203–238.

81. David, J., Maurice, C., Simard, M. 2008. Datations isotopiques effectuees dans le NE de la Province du Superieur – Travaux de 1998, 1999 et 2000. *Ministere des Ressources naturelles* *et de la Faune* 2008; Quebec, DV-2008-05.
